# BMAL1 knockout macaque monkeys display reduced sleep and psychiatric disorders

**DOI:** 10.1093/nsr/nwz002

**Published:** 2019-01-24

**Authors:** Peiyuan Qiu, Jian Jiang, Zhen Liu, Yijun Cai, Tao Huang, Yan Wang, Qiming Liu, Yanhong Nie, Fang Liu, Jiumu Cheng, Qing Li, Yun-Chi Tang, Mu-ming Poo, Qiang Sun, Hung-Chun Chang

**Affiliations:** 1 Institute of Neuroscience, State Key Laboratory of Neuroscience, CAS Key Laboratory of Primate Neurobiology, CAS Center for Excellence in Brain Science and Intelligence Technology, Chinese Academy of Sciences, Shanghai 200031, China; 2 CAS Key Laboratory of Tissue Microenvironment and Tumor, Shanghai Institute of Nutrition and Health, Shanghai Institutes for Biological Sciences, University of Chinese Academy of Sciences, Shanghai 200031, China; 3 Dynamic Brain Signal Analysis Facility, Institute of Neuroscience, Chinese Academy of Sciences, Shanghai 200031, China; 4 College of Life Sciences, University of Chinese Academy of Sciences, Beijing 100049, China; 5 Shanghai Research Center for Brain Science and Brain-inspired Technology, Shanghai 200031, China

**Keywords:** circadian rhythms, BMAL1, macaque monkey, sleep disruption, psychosis, aging

## Abstract

Circadian disruption is a risk factor for metabolic, psychiatric and age-related disorders, and non-human primate models could help to develop therapeutic treatments. Here, we report the generation of BMAL1 knockout cynomolgus monkeys for circadian-related disorders by CRISPR/Cas9 editing of monkey embryos. These monkeys showed higher nocturnal locomotion and reduced sleep, which was further exacerbated by a constant light regimen. Physiological circadian disruption was reflected by the markedly dampened and arrhythmic blood hormonal levels. Furthermore, BMAL1-deficient monkeys exhibited anxiety and depression, consistent with their stably elevated blood cortisol, and defective sensory processing in auditory oddball tests found in schizophrenia patients. Ablation of BMAL1 up-regulated transcriptional programs toward inflammatory and stress responses, with transcription networks associated with human sleep deprivation, major depressive disorders, and aging. Thus, BMAL1 knockout monkeys are potentially useful for studying the physiological consequences of circadian disturbance, and for developing therapies for circadian and psychiatric disorders.

## INTRODUCTION

The circadian clock governs multiple physiological activities, including the diurnal cycle, body temperature, metabolic rhythms, feeding and sleep [1–3]. Experimental disruption of circadian rhythm in flies and rodents results in phenotypes resembling symptoms associated with numerous diseases, such as sleep disorders, cardiovascular dysfunction, diabetic mellitus, cancer and neurodegenerative diseases [1,4,5]. Notably, all of these are chronic conditions that are highly correlated with aging progression [1,4,6,7]. Many clinical studies have identified circadian gene mutations as vital causative factors for psychiatric disorders [8,9]. However, due to limited phenotypes found in standard rodent models, particularly those related to psychiatric disorders, the molecular basis underlying the circadian–psychosis link remains unclear. To recapitulate clinical conditions associated with circadian rhythm dysfunction, diurnal animals with behavioral and metabolic properties closer to humans are highly desirable. Non-human primates thus represent the best choice for this purpose [10,11]. With the rapid advances in gene-editing technologies, the genetic manipulation of non-human primates has become feasible [12,13], and methods for shortening the long reproductive cycle of non-human primates have also been developed [14]. Several transgenic and gene-edited non-human primates have been generated as potential animal models for metabolic stress, neurodegenerative diseases, immunodeficiency, autism and perinatal lethality [12,15–19].

Brain and Muscle ARNT-Like 1 (BMAL1) is a key component of the CLOCK-BMAL1 transcription factor complex, which activates the expression of a great majority of circadian genes. Deletion of BMAL1 in mice results in strong arrhythmic phenotypes, glucose intolerance and premature aging [20–22]. In this study, we ablated BMAL1 in cynomolgus monkeys (*Macaca fascicularis*) and observed arrhythmic circadian activities in pre-adolescent monkeys, with altered rapid eye movement (REM) and non-REM (NREM) sleep, disrupted circadian cycling of many hormones, as well as behaviors resembling anxiety, depression and schizophrenia in humans. Together with results from transcriptional network analysis, our findings provide a direct molecular link between circadian dysregulation and multiple diseases, and indicate the usefulness of non-human primates as animal models for the study of pathogenic mechanisms underlying circadian-related disorders and for developing intervention strategies.

## RESULTS

### *BMAL1* editing in cynomolgus monkeys

**Figure 1.**
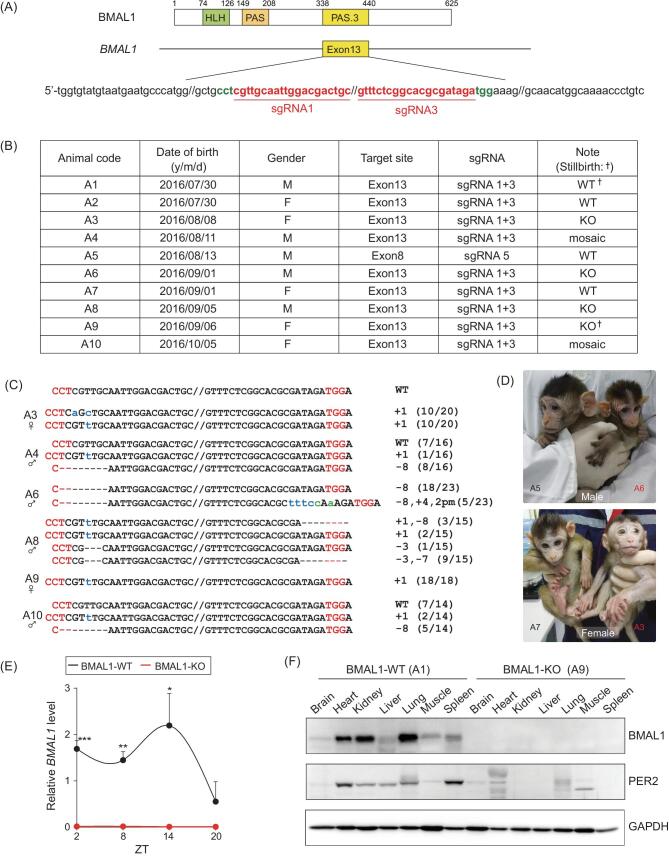
Generation of BMAL1 knockout cynomolgus monkeys. (A) Designated PAS.3 domain and the respective sequences in *BMAL1* exon 13 that were targeted in cynomolgus monkeys. Red, sgRNA1 and 3 sequences. (B) Summary of all *BMAL1*-edited monkeys in this study. (C) Sequence alterations in the five CRISPR/Cas9-edited mutant monkeys, examined via PCR amplification of *BMAL1* exon 13, followed by sequence analysis. (D) Images of male A5 (BMAL1-WT), male A6 (BMAL-KO), female A7 (BMAL1-WT), and female A3 (BMAL1-KO) cynomolgus monkeys at 4 months of age. (E) Expression of WT *BMAL1* transcript over zeitgeber time (ZT) 2, 8, 14 and 20 for BMAL1-WT versus BMAL1-KO monkeys, shown by average blood mRNA levels. (F) Immunoblots of BMAL1 and PER2 levels in major tissues collected from stillbirths A1 and A9. GAPDH was used as the reference. *P < 0.05, **P < 0.01 and ***P < 0.001; Student's *t*-test.

To generate *BMAL1*-edited macaque monkeys, we first tested a battery of single guide RNAs (sgRNAs) targeting exons 8, 10 or 13 of the *BMAL1* locus in cultured embryonic monkey cell lines. We found that a combination of sgRNA1 and 3 (Fig. 1A and Table S1), which target exon 13 that encodes a crucial PAS domain for BMAL1 transcriptional activity, was the most efficient site for *BMAL1* editing. We then performed clustered regularly interspaced short palindromic repeats (CRISPR)/Cas9 editing of cynomolgus zygotes, which were obtained by intracytoplasmic sperm injection (ICSI), to target *BMAL1* exon 13 with sgRNA1 and 3. For comparison, we also edited a separate group of zygotes using sgRNA5 targeting exon 8. In total, 88 edited embryos were transferred to 31 surrogate recipient monkeys and 10 pregnancies were obtained, yielding eight healthy live births and two spontaneously aborted fetuses (Fig. 1B). Among the eight live animals, five showed mutated *BMAL1* and three exhibited wild-type *BMAL1* (wild type, ‘WT'), as indicated by polymerase chain reaction (PCR) analysis of skin samples. *BMAL1* was found to be mutated in all examined skin cells in female A3 and males A6, A8 (knockout, ‘KO'), with mutations involving base pair deletion (‘−'), insertion (‘+') and point mutation (‘pm') (Fig. 1C). In contrast, A4 and A10 were partially edited for *BMAL1* (‘mosaic') (Fig. 1B–D). The subsequent whole-genome sequencing followed by off-target PCR analysis from blood samples demonstrated that *BMAL1* editing was specific in this cohort (Tables S1 and S2). Peripheral blood mRNA analysis confirmed the complete loss of the *BMAL1* transcript in BMAL1-KO monkeys A3, A6 and A8, resulting in down-regulation of several core clock genes as expected (Fig. 1E and Fig. S1). Immunoblot analysis on aborted fetuses A1 and A9 also revealed a complete absence of BMAL1 and consequent PER2 down-regulation in multiple tissues (Fig. 1F), consistent with the anticipated circadian disturbance at the molecular level.

### Abnormal nocturnal activity and blood hormones in BMAL1-KO monkeys

**Figure 2.**
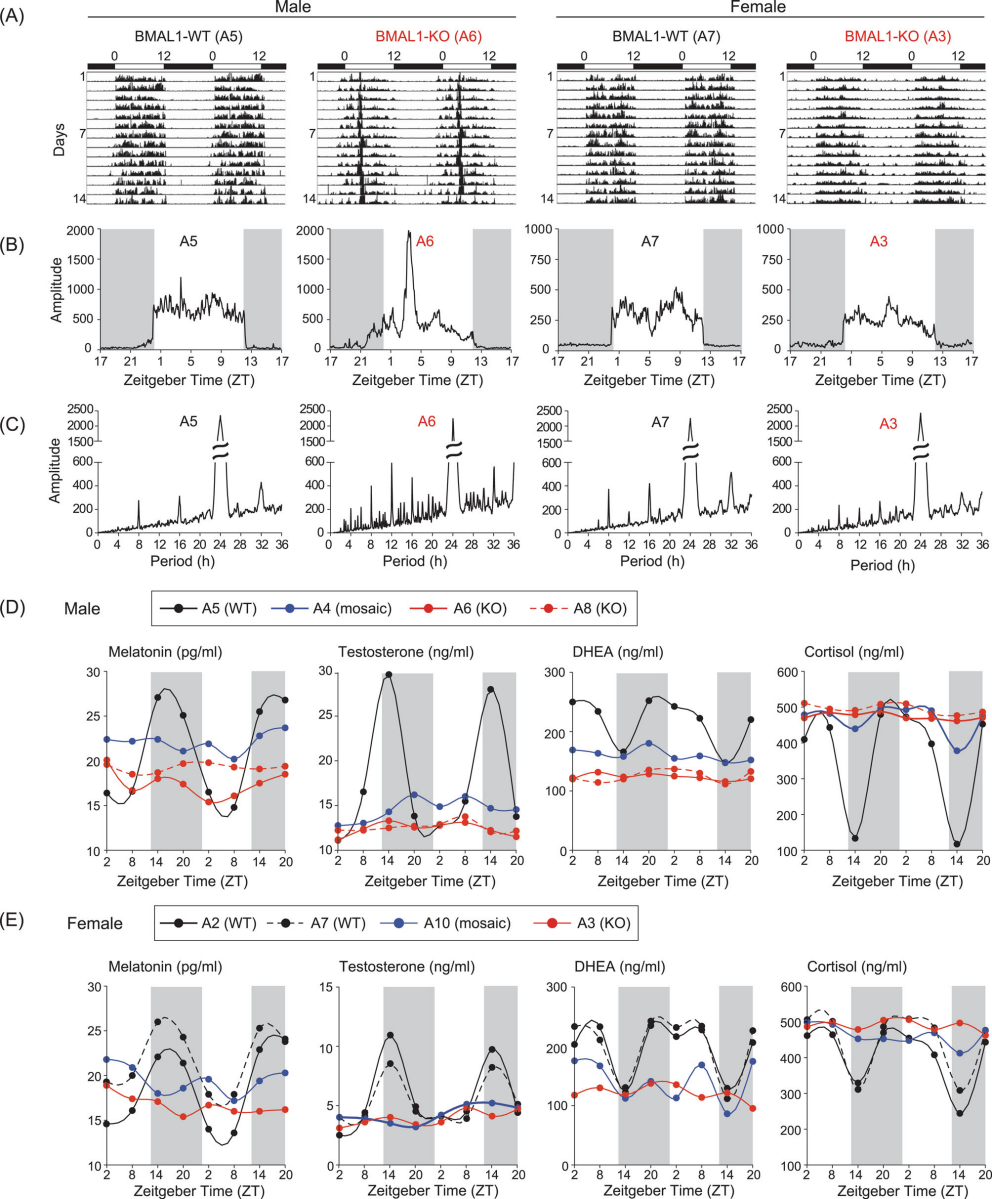
Dysregulated circadian locomotor activities and hormonal levels in BMAL1-deficient monkeys. (A) Continuous 14-d locomotor activity recorded in BMAL1-WT and BMAL1-KO monkeys. Example actograms of A5, A6, A7 and A3 are shown under L/D conditions. (B and C) Activity amplitude and chi-squared periodograms of the 14-d actograms. (D) Levels of melatonin, testosterone, DHEA and cortisol were assayed for monkey plasma samples obtained from temporal collections at 6-h intervals over 48 h. Results shown are from the male cohort. (E) Female hormonal assays in the same manner as in (D).

To study the locomotor activity of *BMAL1*-edited monkeys, we tracked their voluntary locomotion with telemetric actimeters under 12-h light/12-h dark conditions (L/D) for 14 d (see Methods). We found that BMAL1-KO monkeys displayed an increased nocturnal active phenotype at 10 months after birth (Fig. 2A), particularly in the male monkey A6. As summarized for all 14 d (Fig. 2B), we observed higher overall nocturnal locomotor activities in both A6 and A3 monkeys, as compared to BMAL1-WT monkeys, with marked elevation of activities during late night in A6. Periodical analysis also revealed circadian rhythm irregularity, with BMAL1-KO animals showing multiple circadian periods (Fig. 2C). The results supported the conclusion that BMAL1 deficiency caused circadian locomotor abnormality, as previously found in *Bmal1* KO mice [20].

Daily cyclic release of neuroendocrine hormones is vital to support circadian-dependent downstream effector activities [23]. Dampened cyclic levels of hormones, particularly melatonin, were considered a cause of circadian dyssynchrony with aging [24]. Due to the strong association of circadian-related neuroendocrine factors with healthy physiology, we examined the rhythmic change of plasma melatonin levels, which normally peaks at early night and drops to the lowest level during mid-day. By assaying blood melatonin levels every 6 h over 2 d, we found that control monkeys (A2, A5 and A7) showed normal rhythmic melatonin expression, but that BMAL1-deficient monkeys—including A3, A6 and A8 (BMAL1-KO), as well as BMAL1 mosaic A4 and A10—all showed lower melatonin levels without obvious rhythmicity (Fig. 2D and E), reminiscent of that found in aged rhesus monkeys [25]. The levels of testosterone and dehydroepiandrosterone (DHEA) were also higher and rhythmically expressed in control monkeys, but greatly dampened to low levels in BMAL1-deficient monkeys, again resembling aging-related disturbance of these hormones in rhesus monkeys [26]. Notably, we found that the usual cortisol decline at early night in WT monkeys was essentially absent in BMAL1-deficient monkeys (Fig. 2D and E). As shown later, this stably elevated cortisol level is consistent with the findings of behavioral phenotypes resembling psychiatric disorders [27].

### Disrupted sleep states and diurnal EEG oscillations in BMAL1-KO monkeys

**Figure 3.**
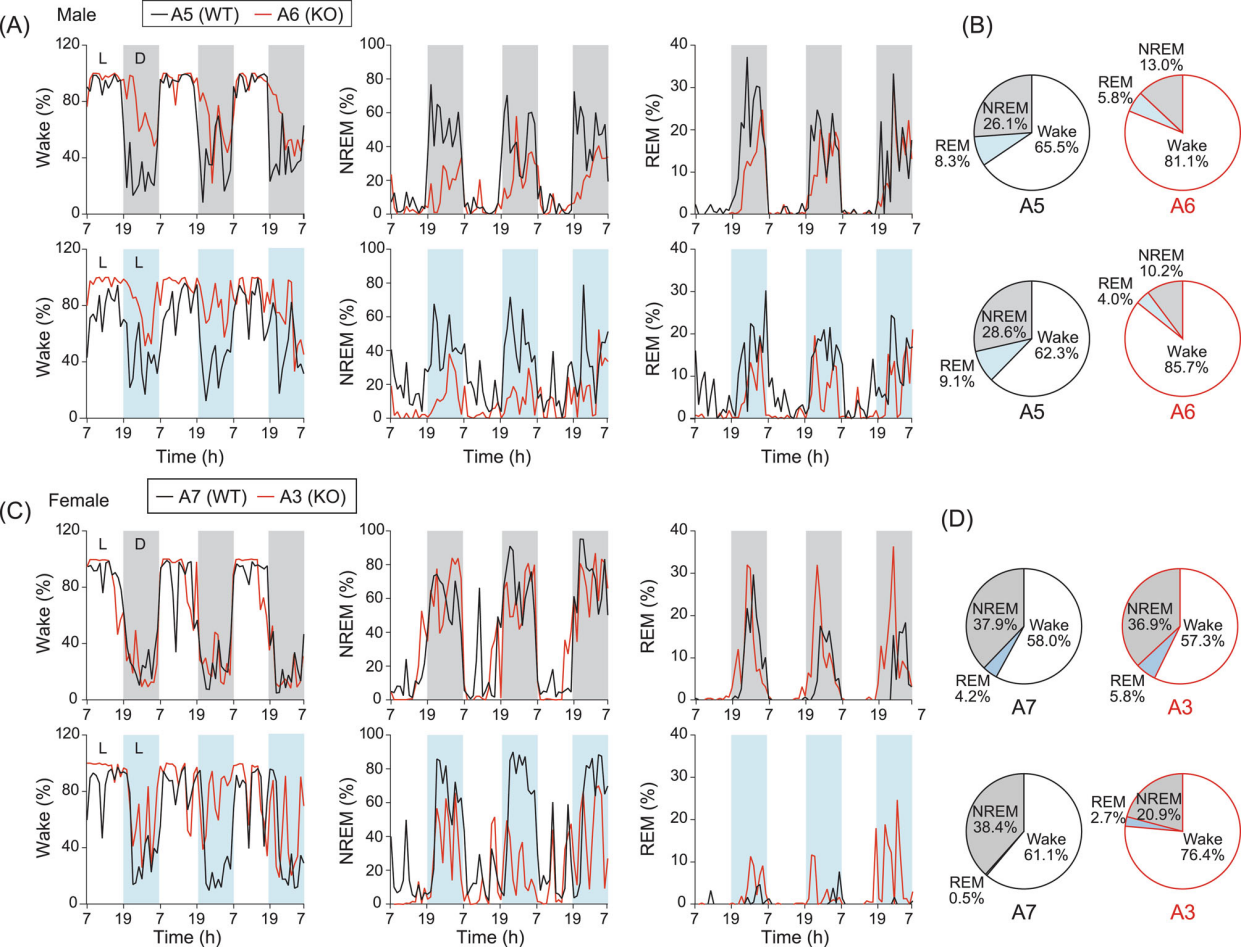
Altered sleep states and diurnal EEG oscillations in BMAL1-KO monkeys. (A) Telemetric EEG recoding in male BMAL1-WT (A5) and BMAL1-KO (A6) monkeys under 3-d L/D cycles (upper panels), or 3-d L/L cycles (lower panels). Stage scores for the proportions of awake, NREM and REM states are shown. (B) Pie charts presented the averaged proportions of the three stages over the 3-d recording period in male monkeys. (C) Telemetric EEG recoding in female BMAL1-WT (A7) and BMAL1-KO (A3) monkeys under 3-d L/D cycles (upper panels) and 3-d L/L cycles (lower panels). (D) Pie charts are presented as in (B) for female monkeys.

The disrupted melatonin levels together with arrhythmic locomotor activity found in BMAL1-KO monkeys suggested an imbalanced sleep homeostasis. Indeed, by analyzing the sleep/awake state-specific electroencephalography (EEG) power spectra, we found that monkey A6 under normal L/D conditions exhibited a higher awake state, as well as lower REM and NREM states, at night, as compared to those in control monkey A5. The reduced sleep time in monkey A6 is opposite to that found in *Bmal1* KO mice, implicating that major differences in BMAL1 regulation may exist between diurnal and nocturnal animal models [28]. These differences were even more pronounced for monkey A6 under 3-d constant light illumination (L/L), which was designed to examine the internal circadian maintenance in response to disturbed external cues (Fig. 3A and B) (constant dark conditions were not practical in our study due to animal care requirements). For the awake and sleep states, we did not find obvious differences between female monkey A3 and the control monkey A7 under the normal L/D conditions, but clear disruption was observed in A3 under L/L conditions, especially during the second and third day of L/L exposure (Fig. 3C and D). The awake/sleep EEG data are summarized by pie charts in Fig. 3 (B and D) and Table S3. Rhythmic changes of body temperature are often used as a functional index of circadian rhythm. We found that fluctuations in body temperature in BMAL1-KO monkeys were much larger than those in control monkeys, particularly under the L/L conditions. The daily cycle of body temperature fluctuations persisted in control monkeys, but was essentially abolished in both BMAL1-KO monkeys on days 2 and 3 after switching to the L/L conditions (Fig. S2).

### Psychosis-like behavioral phenotypes in BMAL1-deficient monkeys

**Figure 4.**
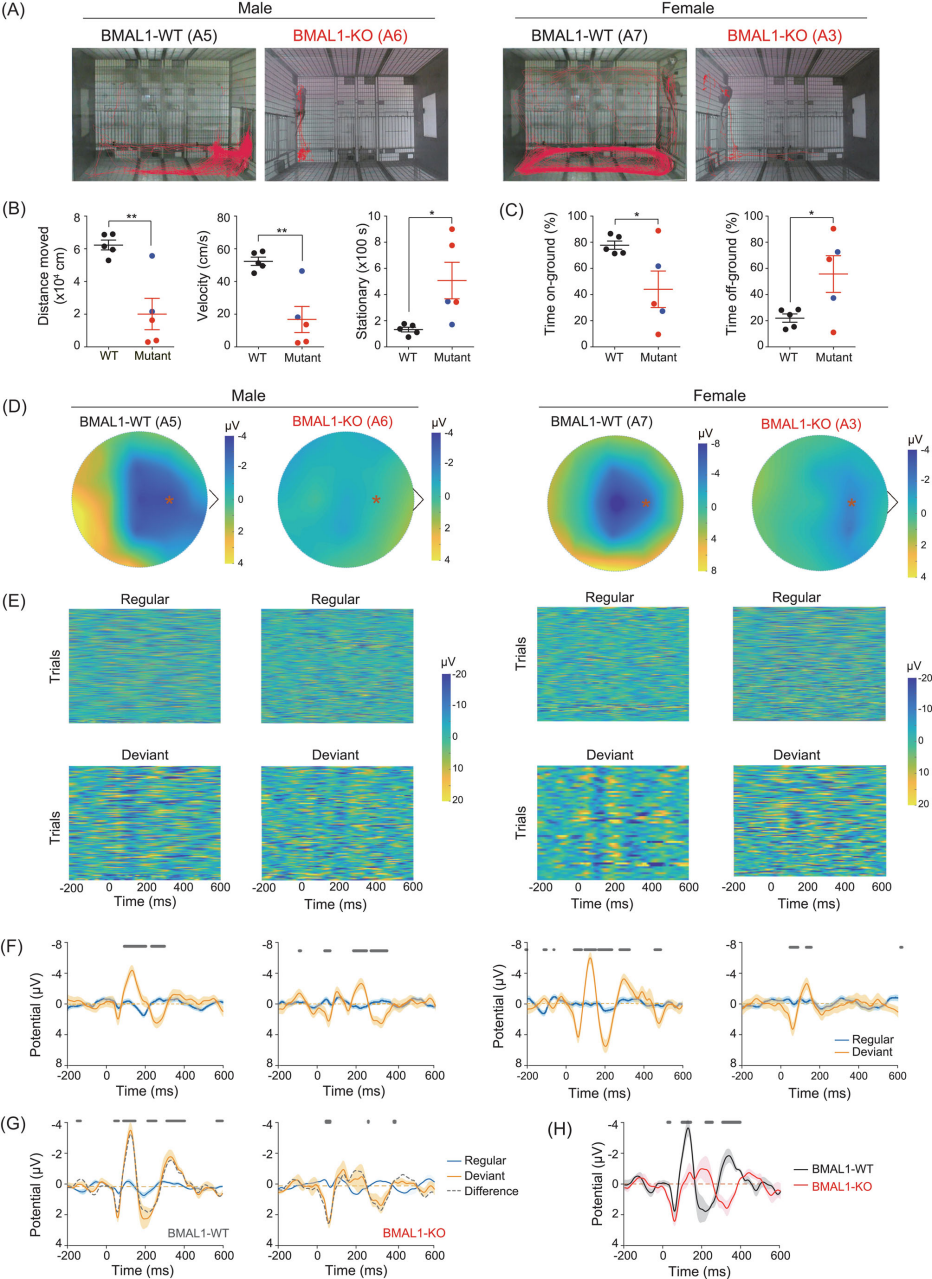
Depression-related behavior and sensory processing impairment in BMAL1-KO monkeys. (A) Activity tracks (marked red) obtained by 20-min videotape recordings for all BMAL1-edited and WT monkeys. (B) Summary of locomotor activities, including total distance, moving velocity, stationary time, and (C) time spent on-ground versus time spent off-ground were analyzed. Black, blue and red indicate BMAL1-WT, BMAL1 mosaic and BMAL1-KO monkeys, respectively. Data also include two WT age-matched male monkeys, in addition to the eight monkeys listed in Fig. 1B. *P < 0.05 and **P < 0.01; Student's *t*-test. (D) Topographic voltage maps of BMAL1 WT and KO monkeys. Asterisk indicates Fz electrode position. (E) Heat maps of voltage amplitudes induced by regular or deviant sound trials. ERPs at Fz electrode were analyzed. (F) Average ERP summarized from (E). (G) MMN of BMAL1 WT and KO monkeys, as indicated by the differences between regular and deviant in dashed lines. (H) MMN of BMAL1-WT versus BMAL-KO monkeys summarized from (G). Significant differences are indicated with star labels on top (*P* < 0.05, Student's *t*-test), and SEMs are shown as shaded areas.

The constant high-level cortisol implicates a potential predisposition to stress and depression [29]. Indeed, videotape behavior tracking of BMAL1-deficient monkeys in a novel environment (of a new cage) revealed several abnormalities. First, in contrast to the active exploration of control monkeys, BMAL1-deficient monkeys remained largely stationary over the 20-min recording period, indicative of a stressed condition (Fig. 4A and B) [30]. Furthermore, the area covered by locomotor activities was restricted to corners away from the corridor, and the time spent off-ground were significantly higher (Fig. 4C), reminiscent of the reduced cage exploration of monkeys due to stress [31]. These phenotypes were most pronounced in BMAL1-KO monkeys A6 and A3, as illustrated in Fig. 4C (see Movies S1–S4). Monkey A6 exhibited particularly strong fear and anxiety, showing clear avoidance of the care personnel in his home cage by retreating to a cage corner and burying his head in his hands (Movies S5 and S6). Notably, the latter behaviors were not observed when A6 was undisturbed, suggesting that this abnormality was triggered by external inputs. When these BMAL1-deficient monkeys were re-exposed to the same cage 1 month later, we observed similar phenotypes of a higher tendency to remain stationary and staying off-ground when moved, as compared to control monkeys (Fig. S3). These behaviors are consistent with neuroendocrine dysfunctions, as exemplified by the persistently elevated cortisol level that is known to be associated with depression and anxiety [29,32].

Using an auditory oddball test, which measures perception of infrequent changes in normal sound sequences as reflected by mismatch negativity (MMN) responses in event-related potential (ERP) [33], we found that BMAL1-KO monkeys A6 and A3 were defective in MMN responses. The average peak MMN amplitudes after the deviant stimulus onset (range 100–150 ms) were significantly lower than those found in BMAL1-WT A5 and A7, as shown by the example heat maps of ERP amplitudes over the entire scalp mapped with 21 EEG electrodes (Fig. 4D). Roster plots of ERP amplitude for 60 deviant trials recorded by a Fz electrode (marked by red * in Fig. 4D) showed reduced repeatability of MMN in ERPs induced by deviant stimuli in BMAL1-KO monkeys (Fig. 4E), as also presented by the summary plots of ERPs during 240 regular versus 60 deviant trials (Fig. 4F). The average ERPs of control and BMAL1-KO monkeys evoked by regular and deviant sounds (Fig. 4G), and the difference of regular versus deviant ERPs for two types of monkeys (Fig. 4H), both showed marked reduction in MMN amplitudes for the BMAL1-KO monkeys. The impaired cognitive function in novelty detection suggests BMAL1 deficiency had triggered a schizophrenia-like symptom [34]. Taken together, the behavioral and electrophysiological results indicated that BMAL1-KO monkeys represent a potential non-human primate model for studying the causal link between circadian disorders and psychosis.

### Transcriptome analysis revealed inflammatory dysregulation and chronic maladies

**Figure 5.**
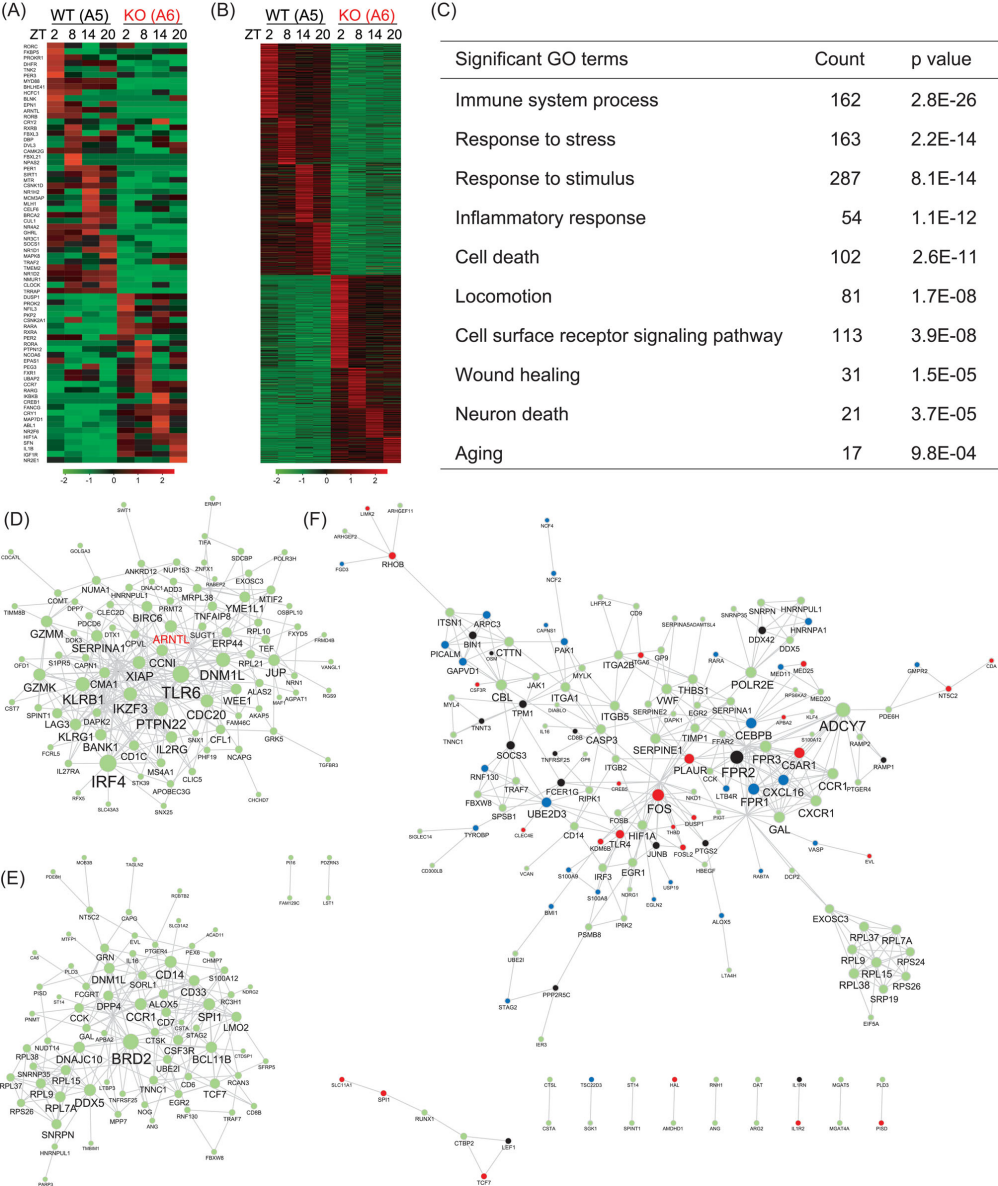
Blood transcriptome analysis of BMAL1-KO monkeys. (A) Representative examples of the expression levels of circadian controlled genes over time points ZT2, ZT8, ZT14 and ZT20. Results from male BMAL1-WT (A5) and BMAL1-KO (A6) monkeys are shown. (B) Heat maps of genes that were up- and down-regulated in their expression over time points ZT2, ZT8, ZT14 and ZT20 for monkeys A5 and A6. Results of greater than twofold changes are shown. (C) GO terms for up-regulated genes shown in the transcriptome analysis in (B). (D and E) STRING assembly for functional network in A5 (D, upper panel) and A6 (E, lower panel) at time point ZT2. The result shows that the network assembly became distinctly different between WT and BMAL1-KO monkeys (BMAL1 is labeled as ‘ARNTL' in the network). (F) Over-representing STRING network assembly of up-regulated targets in BMAL1-KO A6. Targets related to sleep deprivation (GSE39445) and major depressive disorder (GSE76826) are shown in black and blue, respectively, and those related to both are shown in red.

To examine the impact of BMAL1 ablation on transcriptional activities, we carried out a temporal analysis of blood samples from control monkey A5 and BMAL1-KO monkey A6 for RNA sequencing at different time points (zeitgeber time (ZT) 2, 8, 14 and 20). As expected, we found altered expression of a large number of circadian-controlled transcripts in A6 samples, mostly showing reduced expression (Fig. 5A). Among core circadian genes, *RORβ*, *PER1* and *NPAS2* also exhibited the absence of circadian phasic expression (Fig. 5A). Among 1402 transcripts identified with twofold expressional changes between A5 and A6 (Fig. 5B and C, and Table S4), we noticed a group of inflammatory targets that were elevated and dysregulated throughout all four time points, including *IL1R2* and *NFKBIZ*, suggesting that systemic inflammation was induced by circadian disruption (Fig. 5B and C, and Table S4). Gene ontology (GO) analysis of up-regulated genes also showed that immune system processes and inflammatory responses were high on the list (Fig. 5C). This is consistent with the findings that the immune system is rhythmically regulated along with the circadian program for its activation and suppression homeostasis [35], and that BMAL1 plays a suppressive role in inflammatory monocyte populations [36]. Other biological processes revealed in GO terms included stress responses, response to stimulus and locomotion (Fig. 5C and Table S5), correlating well with psychiatric phenotypes described above for BMAL1-KO monkey A6.

A transcriptome assembly depicted by STRING indicated a distinct functional network activated by BMAL1 ablation. Re-assigned programs with up-regulated clusters such as *BRD2*, *DPP4* and *DDX5* implicate inflammation, metabolic imbalance and predisposition to oncogenic risks, respectively (Fig. 5D and E). To further explore the potential dysfunctions connected to circadian disorders due to BMAL1 deficiency, we compared blood transcriptome databases for human subjects with sleep deprivation (GSE39445) [37], major depressive disorder (GSE76826) [38] and aging (GSE75337) [39]. We found significant correlations between up-regulated genes in A6 and those associated with the above three human conditions. Many genes, e.g. *FOS*, *DUSP1* and *HNRNPA1*, were up-regulated in more than one condition, suggesting potential molecular links between sleep deprivation and mood disorders (Fig. 5F). Perhaps the most interesting genes are those up-regulated in all three conditions, such as *TLR4* and *CREB5*, which may serve as vital triggers for pathways that link circadian disorders to chronic diseases and aging (Fig. 5F and Fig. S4). Together, these results suggest that the macaque model described here may be useful for probing cellular and molecular mechanisms underlying circadian-related disorders.

## DISCUSSION

Progress in translating basic research findings to treatments of human diseases has often been limited by the availability of animal models that mimic symptoms of human disorders. A suitable model for translational purposes should be complex enough to display as many phenotypes as those observed in humans, and exhibit physiological and anatomical features close to human. For example, the intrinsic difference in diurnal versus nocturnal activity patterns may blur the effects of external perturbation, e.g. the L/D cycle, on molecular oscillations and interfere with the design of therapeutic treatments. A recent diurnal transcriptome atlas derived from baboon experiments indicated distinct phases of rhythmic gene expression from those found in mice [40], suggesting the need for non-human primate models beyond nocturnal rodents. The present BMAL1-deficient monkey is a preferred model for circadian disruption for the following reasons. First, BMAL1 exerts crucial activity in transcriptionally regulating the circadian program in most tissues, thus the ablation offers opportunities to investigate both the central and peripheral clocks with minimal concern about genetic redundancy. Second, BMAL1 KO in mice causes circadian arrhythmicity, sleep and metabolic disorders [4,41], and premature aging [22,42], and a non-human primate model could be useful for multiple disease areas in supplement of deficiencies found in *Bmal1* mice. Finally, BMAL1 has been linked to psychiatric conditions [8,9]. Studies on the effects of BMAL1 ablation in a monkey model, together with appropriate behavioral assays, could help us to understand the molecular basis of the circadian link to mood disorders.

Useful rodent models have been developed for the study of circadian dysfunction and mood disorders [43], including suprachiasmatic nucleus disruption [44], and *Clock-Δ19* and *Per1^Brdm1-/-^* mutations [45,46]. The monkey model described here offers additional observations on behaviors relating to psychiatric disorders. The stressed behaviors seen in BMAL1-KO monkeys, such as the head being buried in the hands (Movie S6), avoiding care personnel and schizophrenia-related cognitive impairment in auditory oddball MMN trials, are rather unique among animal models. Further interventional studies aiming at ameliorating specific behavioral abnormalities in BMAL1-deficient monkeys will be useful for the development of psychiatric treatments.

The transcriptome results indicated over-representation of immunological and inflammatory responses upon BMAL1 ablation, consistent with the findings that several immune mediators are affected by circadian oscillations, thus weakening clock programs can lead to immune dysregulation [47], including abnormal regulation of the toll-like-receptor 4/tumor necrosis factor α pathway in myeloid cells [48] and the activation of inflammatory monocytes [35,36]. Longitudinal analyses of BMAL1-KO monkeys will help us to understand the pathogenesis of and develop the therapeutics for inflammatory chronic diseases induced by circadian dysfunctions.

## METHODS

### Animal ethics statement

The use and care of cynomolgus monkeys (*M*. *fascicularis*) complied with the guidelines of the Animal Advisory Committee at the Shanghai Institutes for Biological Science, Chinese Academy of Sciences, under the approval application entitled ‘Reproductive physiology of cynomolgus monkeys and establishment of transgenic monkeys'(ER-SIBS-221106P). The monkeys in the experiment were housed in a conditioned environment (temperature: 22 ± 1°C; humidity: 50% ± 5%) with a 12-h L/D cycle (light on time 07:00 to 19:00). All animals were fed a commercial monkey diet (Anmufei, Suzhou) twice a day with free access to tap water, and with fruits and vegetables supplemented once daily. Animals were been under careful veterinary surveillance to ensure health conditions during and after experiments.

### Superovulation, oocyte collection and gene editing

Healthy female cynomolgus monkeys with regular menstrual cycles were chosen for superovulation and oocyte collections were carried out via laparoscopy. From day 3 of the menstrual cycle, 25 IU recombinant human follitropin was injected intramuscularly twice daily for 7–8 d. On day 11, 1000 IU of human chorionic gonadotrophin was administrated, followed by oocyte collection from follicles (2–8 mm in diameter) 36 h later. The collected oocytes were cultured in pre-equilibrated hamster embryo culture medium 9 (HECM-9) medium. Metaphase II-arrested oocytes were selected for further manipulation [49]. *Cas9* and sgRNA mRNAs were prepared as previously reported [50]. For sgRNA preparation, a T7 promoter containing a specific forward and a common reverse primer was used to amplify the sgRNA template by PCR, and the resulting PCR product was used for *in vitro* transcription using a MEGAshortscript T7 kit (Thermo Fisher Scientific). For *Cas9* mRNA, a T7 promoter containing a specific forward and a common reverse primer was used to amplify the *Cas9* coding region, and the resulting PCR product was used for *in vitro* transcription using an mMESSAGE mMACHINE T7 ULTRA kit (Thermo Fisher Scientific). *Cas9* mRNA and sgRNA purifications were achieved using a MEGAclear kit.

Fertilization was performed via ICSI [49], and the presence of two pronuclei and two polar bodies was confirmed. The CRISPR/Cas9 method was applied for *BMAL1* (gene ID 101865448) gene editing in the zygotes. The design of sgRNA followed the instructions on the website of the Zhang laboratory at the Broad Institute (http://crispr.mit.edu/job/9950152458235442), and the sequences are listed in Fig. 1A and Table S1. Approximately 5 pl of 50 ng/μl sgRNA and 100 ng/μl *in vitro*-transcribed *Cas9* mRNA were mixed and injected in the cytoplasms of fertilized oocytes. Injected embryos were cultured in HECM-9 media containing 5% fetal bovine serum at 37°C in 5% CO_2_ to allow embryo development. Next, 31 menstruation-synchronized females were used as surrogate recipients for sgRNA-*Cas9*-injected embryos. Typically, two pronuclear–early-stage embryos were selected for tubal transfer to each surrogate female [49].

### Genotyping analysis

Tissues, including ear skin fibroblasts and peripheral blood from founder monkeys, were collected and digested in lysis buffer (10 mM Tris-HCl, 0.4 M NaCl, 2 mM ethylenediaminetetraacetic acid (EDTA), 1% SDS and 100 mg/ml Proteinase K) overnight at 65°C. The genomic DNA was then purified by phenol–chloroform extraction and alcohol precipitation. PCR was performed using targeted gene-specific primers BMAL1-F: 5′-TGGTGTATGTAATGAATGCCCATGG-3′ and BMAL1-R: 5′-TAGAGACAGGGTTTTGCCATGTTGC-3′, and PCR products were then sub-cloned into pMD19-T vector for sequencing and verification of the mutations, as shown in Fig. 1C.

### Off-target analysis

The genomes of CRISPR/Cas9-edited monkeys were sequenced using the Illumina NovaSeq 6000 System (Illumina, San Diego, CA, USA). Qualified reads were mapped to the assembly *M*. *fascicularis* genome (v5) using BWA (v0.7.12-r1044) and the variants/indels (insertion/deletions) were identified using Sentieon Genomics (ver.201611.02). The potential off-target sites were predicted using a genome-wide CRISPR/Cas9 off-target site prediction tool Cas-OFFinder (http://www.rgenome.net/cas-offinder/) [51]. We first predicted the off-target sites with conditions that allowed three to five mismatches to occur in the sgRNA sequences of sgRNA1, 3 and 5. We further searched for single nucleotide polymorphisms (SNPs)/indels that occurred in the 5 bp-spanning range at the predicted sequences. Cases with SNPs/indels occurring in the analysis were further subjected to PCR and Sanger sequencing validation using blood DNA samples, together with respective parental PCR sequencing assays to ascertain the sources of sequence variations. The off-target analysis results and related primer information are summarized in Tables S1 and S2, respectively.

### Immunoblotting

Brain, heart, kidney, liver, lung, muscle and spleen tissues were carefully removed from stillbirth carcasses of A1 and A9, and stored at −80°C until use. Approximately 50 mg tissue samples were homogenized and lysed in ice-cold lysis buffer (50 mM Tris at pH 7.5, 150 mM NaCl, 1% Triton X-100, 0.5% NP-40 and 10% glycerol) supplemented with complete protease inhibitor (Roche). Protein levels were standardized using the Bradford protein assay before being boiled in Laemmli buffer. Protein samples (25 μg) were resolved in a 10% acrylamide gel then transferred to polyvinylidene difluoride (PVDF) membrane, followed by standard protocols for immunoblotting with the following primary antibodies: rabbit anti-BMAL1 (CST14020S, Cell Signaling Technology), rabbit anti-PER2 (AB2202, Merck Millipore), mouse anti-GAPDH (60004–1-1 g, Proteintech); and secondary antibodies: goat anti-rabbit IgG (H + L)-HRP conjugate (1706515, Bio-Rad) and goat anti-mouse IgG (H + L)-HRP conjugate (1706516, Bio-Rad). Consistent results were obtained from three independent immunoblot sets.

### Blood sample collection

The BMAL1 monkey cohort was studied for blood circadian transcript levels at 12 months of age. Blood hormonal levels and transcriptome analyses were inspected at 12 months of age. Blood samples (0.5 ml) were collected intravenously at time points ZT2 (09:00, 2-h post lights on), ZT8, ZT14 and ZT20 for transcriptional analyses, or the same time points for 2-d continuous sampling for plasma assays. Briefly, blood samples for real-time quantitative PCR or transcriptome assay were pre-incubated with red cell lysis buffer (TIANGEN, RT122–02) to remove erythrocytes, followed by centrifugation at 1200 × g at 4°C for 5 min. Leucocyte pellets were then flash-frozen in liquid nitrogen and stored at −80°C until use for total RNA extraction. Blood samples for plasma assays were first collected into EDTA-coated Improvacuter^®^ vacuum blood collection tubes. The plasma fraction was obtained by centrifugation at 1000 × g for 10 min at room temperature, then stored at −80°C until assay.

### Real-time quantitative PCR

Total RNAs from leucocytes were first extracted using an RNeasy Mini Kit (QIAGEN), followed by cDNA reverse transcription with an Omniscript RT Kit (QIAGEN). Real-time PCR reactions were prepared with the use of a QuantiNova SYBR Green PCR Kit (QIAGEN), then analyzed on a StepOnePlus Real-Time PCR system (Thermo Fisher Scientific). The relative abundance of transcripts was calculated by normalizing to *Med10* level. Six monkeys were analyzed for circadian gene expressions, including three age-matched WT monkeys and the three BMAL1-KOs (A3, A6 and A8). Primers are listed in Table S2.

### Locomotor activity monitoring

Locomotor activity was recorded in monkeys near 10 months of age for 14 d in singly housed condition. Physical activity level was detected with a Xenon ACT II wireless accelerometer (BLEACT; Cloud Care Technologies) that was designed to be lightweight (7 g) and small (4 cm × 1.5 cm × 1.1 cm) for easy, long-term wear purposes [52]. Individual accelerometers were secured in plastic collars, thus facilitated continuous wear for the monkeys. The locomotion activity signals were analyzed with ClockLab Analysis software (Actimetrics, Version 6.0) for the average activity amplitudes over 14 d of recording, and the free-running chi-squared periodogram under a standard 12-h L/D cycle.

### Plasma enzyme-linked immunosorbent assays

Melatonin, testosterone, DHEA and cortisol levels were assayed via enzyme-linked immunosorbent assay kits according to the manufacturers' protocols (BioSource MBS743125, Enzo ADI-900–065, Enzo ADI-900–093 and Enzo ADI-900–071, respectively). Briefly, plasma samples collected from time points were first aliquoted for either direct application in the melatonin assay, or extraction with equal volume of ethyl acetate (DHEA) or diethyl ether (for testosterone and cortisol) for three times. The extracted materials were lyophilized, reconstituted in the respective assay buffers provided in the kits and followed by assaying in a 96-well format plate with incubation, washing and colorimetric measurement steps as described in the manufacturers' protocols.

### Sleep recording and EEG analysis

Wake–sleep activities were analyzed in monkeys near 15 months of age. Sleep recording was carried out by chronically implanting radio telemetry transmitters (PhysioTel Digital M01 implant, Data Sciences International (DSI)) for continuous long-term measuring of EEG and electromyography (EMG) signals. Monkeys were deeply anesthetized by giving an intramuscular dose of 5 mg/kg Zoletil 50 (Virbac S.A.) before implantation surgery. In case where the surgery exceeded 2 h, a supplement of an additional 1 mg/kg Zoletil 50 was provided intramuscularly to maintain deep anesthetization. The DSI implant was embedded subcutaneously in the shoulder–back (at the subscapular region). The EEG electrode biopotential leads were subcutaneously tunneled to the skull, and the two EEG electrodes were screwed into the skull 5 mm lateral, and 5 or 10 mm anterior, respectively, to the lambda midline. Electrode leads for EMG were sutured to the neck musculature. After surgery, monkeys were returned to their home cage with analgesia Ketoprofen (1 mg/kg) provided via intramuscularly dosing daily for 3 d, and antibiotics (Penicillin, 20 MU/kg) provided daily for 1 week. The room for EEG/EMG recording (5.5 × 2.5 × 2.9 m) was equipped with six transceivers (TRX-1) mounted to the walls, thus allowed excellent sampling of telemetric signals transmitted from anywhere in the room. The TRX-1 transceivers were connected to a data-exchange matrix (CLC) then to a computer for recording and data storage. Sleep recording was performed for 3 d under normal illuminating conditions, i.e. lights-on time 07:00–19:00, and then switched to recording under full-day lighting for another 3 d. Wake–sleep-stage scoring was performed with software NeuroScore 3.2.1 (DSI) to identifying stages as awake, REM sleep and slow-wave NREM sleep. The results were depicted every 60 min for the stage proportions for the 3-d period, as shown in Fig. 2. Briefly, EEG signals sampled at 448 Hz were filtered and analyzed by time frequency fast-Fourier transform analysis with a 10-s epoch Hanning window. Gamma (24–100 Hz), beta (16–24 Hz), sigma (12–16 Hz), alpha (8–12 Hz), theta (4–8 Hz) and delta (0.5–4 Hz) frequencies were applied as default NeuroScore 3.2.1 settings for wake–sleep-stage scorings.

### Locomotor behavior observation and analysis

Monkey locomotion behaviors were monitored at the age of 16 months, with video recording during the light cycle in an observation cage (1.5 × 1 × 1.1 m) for 20 min without interruption. The new cage-exploring videos were scored using EthoVision XT software Version 11.5 (Noldus Information Technology). Locomotor activities after tracking were categorized for the results of moving distance, moving velocity and stationary time. The off-ground activity was calculated for the time when all limbs were off the ground.

### Scalp EEG recording and analysis for auditory-intensity oddball paradigm

The passive auditory-intensity oddball paradigm was designed to present 100 ms tones in different intensities (low, 60 dB; high, 100 dB) to monkey subjects. Stimuli of frequent (standard, 240 events, 80% in the recording) and infrequent (deviant, 60 events, 20% in the recording) intensities were presented, with an inter-stimulus interval setting of 700 ms. A 100-dB high-deviant condition was applied in the study. Stimulus presentation was controlled by E-prime 3.0 using a personal computer. Tones were presented using a HP amplifier about 10-cm away from subject ears. An Ag/AgCl electrode, 22-channel EEG cap was applied for scalp EEG recordings, and activity signals were acquired with a Neuroscan SynAmps RT amplifier and Curry 8 software (Neuroscan). EEG data were then analyzed using MATLAB, with data pre-processing procedures including 50 Hz notch filtering, dataset referencing, band-pass filtering and segmentation, prior to ERP calculation. The topographic voltage distribution maps were generated by coordinating all electrodes from three-dimensional positions to a two-dimensional projection by MATLAB. The maps for average potential values reflecting MMN (100–150 ms) are shown.

### RNA sequencing and data analysis

Peripheral blood samples were collected at ZT2, ZT8, ZT14 and ZT20 from BMAL1 monkeys A5 and A6, and total RNAs from leucocytes were extracted using an RNeasy Mini Kit (QIAGEN). The RNA samples were examined with an Agilent 2100 Bioanalyzer to ensure RNA integrity number > 8.0, before being subjected to cDNA library construction and further RNA sequencing analysis. Libraries were prepared using an Illumina TruSeq RNA Library Prep Kit v2, then sequenced via the Illumina Hiseq platform at 150 bp paired-end reads generated. The sequencing data were mapped to the monkey genome (NCBI: *Macaca_fascicularis*_5.0) using bowtie 2 software. Gene expression levels were quantified as fragments per kilobase of exon per million mapped fragments (FPKM) using Cufflinks [53]. For differentially expressed genes between A5 and A6 samples with fold change greater than two, we plotted the gene expression heatmap, performed GO enrichment analysis and constructed the functional association network based on the STRING database (version 10.5). The blood gene expression signatures for sleep deprivation (GSE39445), major depressive disorder (GSE76826) and aging (GSE75337) derived from the Gene Expression Omnibus database were applied to annotate the up-regulated transcripts in BMAL1-KO A6.

### Statistical analyses

We used the Student's *t-*test for the following comparisons: for pairwise comparisons of new cage exploration experiments as shown in Fig. 4A–C and Fig. S3A–C, and for the mean potential values induced by two different tone intensities (regular versus deviant) with time in Fig. 4F–H. Statistical tests were conducted using Prism (GraphPad) or MATLAB.

### Data availability

Data generated during this study are available in the Sequence Read Archive (SRA) repository under accession numbers SRP145518 for the whole-genome sequence, and SRP145029 for RNA sequencing raw data for cynomolgus monkeys. All codes are available upon request.

## Supplementary Material

Supplementary Files
