# Supplementary material for: BMAL1 knockout macaque monkeys display reduced sleep and psychiatric disorders
Source: Natl Sci Rev. 2019 Jan 24;6(1):87–100. doi: 10.1093/nsr/nwz002 (PMC8291534; doi:10.1093/nsr/nwz002)
Supplement: Supplementary Files [file nwz002_supplemental_files.zip › NSR_MS-2018-233.R1_Extended data.docx]

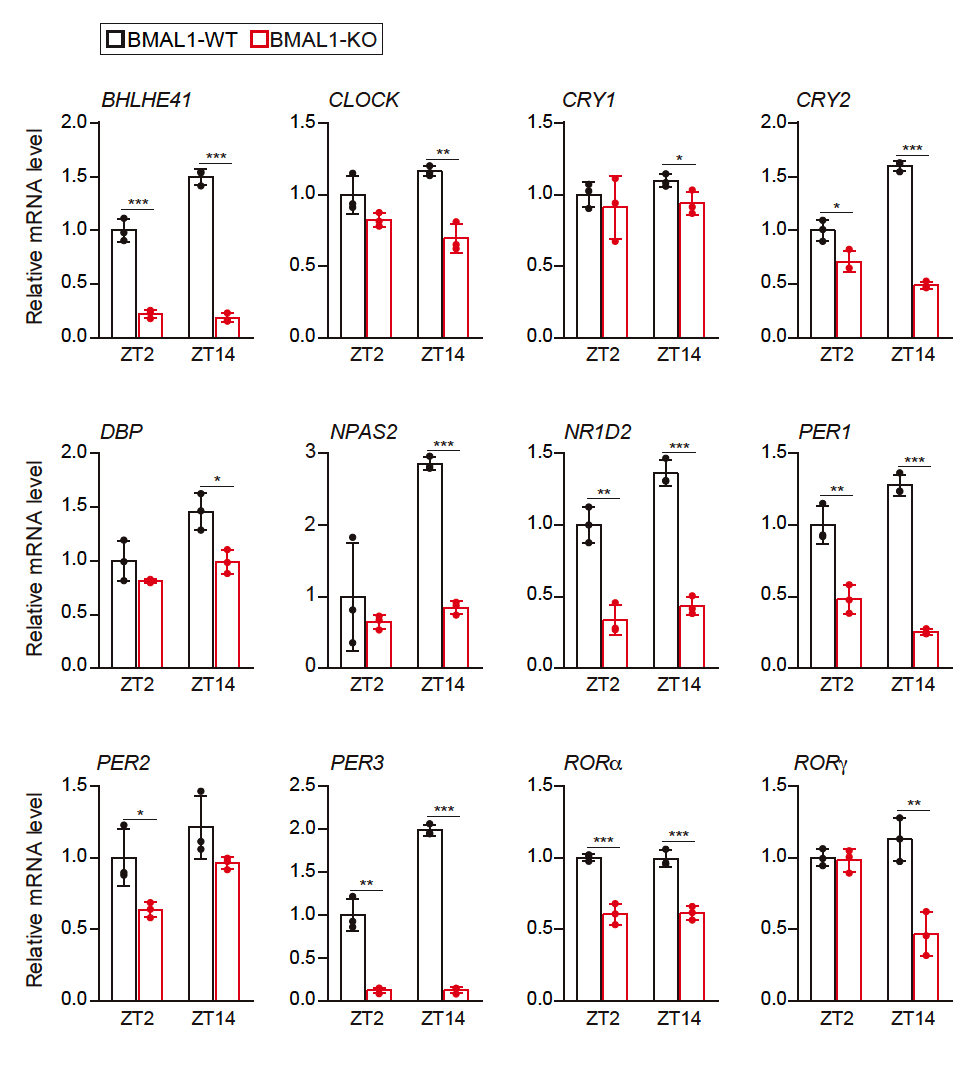


**Figure S1.** Analysis of the expression of core clock genes. Core clock gene transcript analysis in zeitgeber time (ZT) 2 and 14. Average of BMAL1-WT (n=3) *versus* BMAL1-KO (n=3) monkeys were shown. ^🞱^*P* < 0.05, ^🞱🞱^*P* < 0.01, ^🞱🞱🞱^*P* < 0.001; Student’s *t*-test.


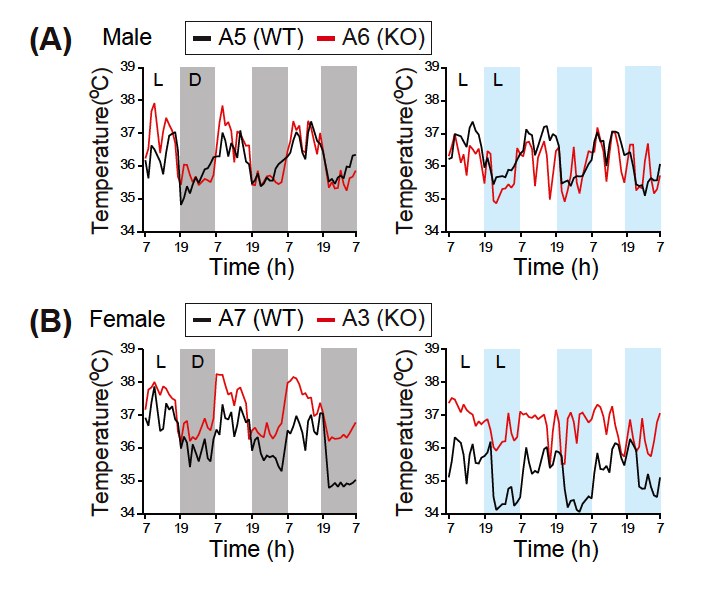


**Figure S2.** Body temperature recordings under 3-day L/D and L/L conditions. (A)(B) Body temperature changes that were recorded under 3-day light/dark or 3-day light/light conditions. Male (A, upper panel) and female (B, lower panel) were shown. Note that the disrupted body temperature cycles occurred in BMAL-KO A6 and A3 became evident during day 2 and day 3.


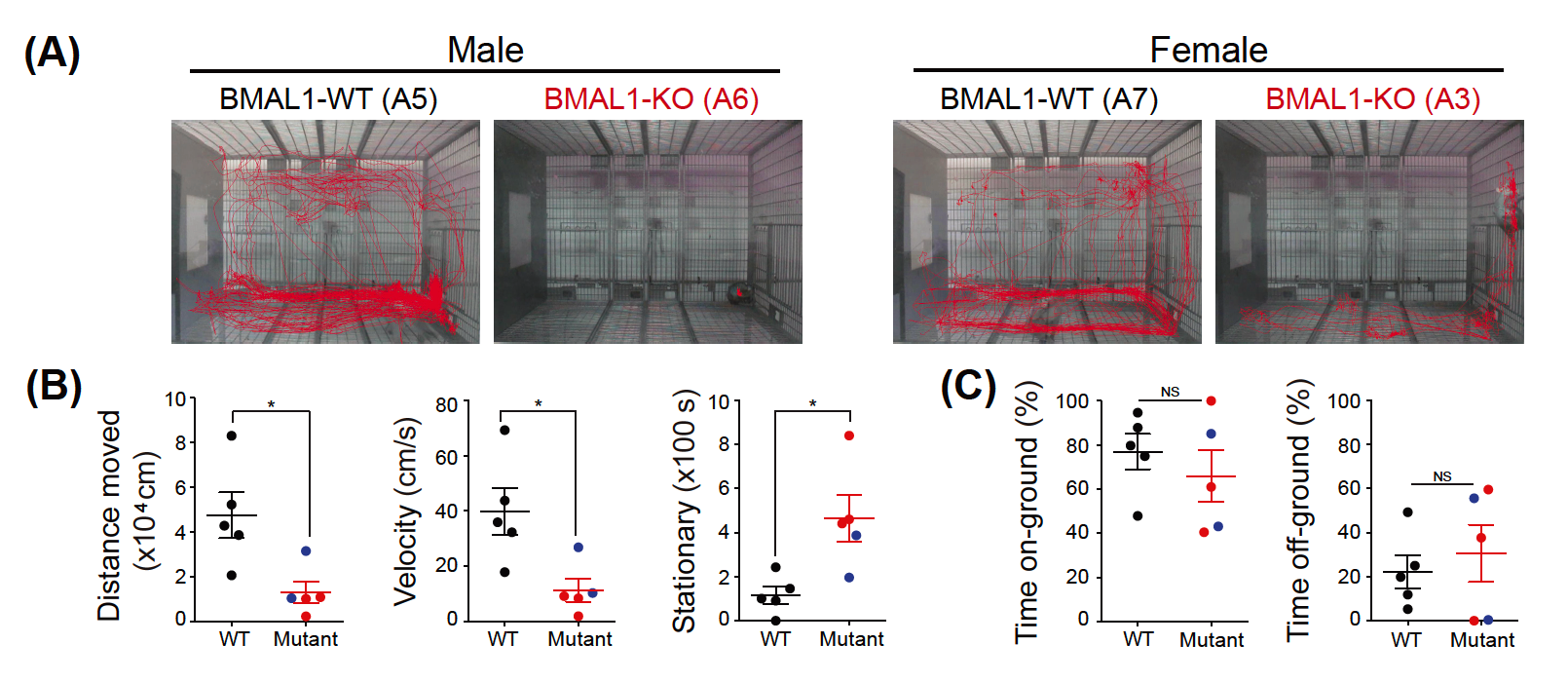


**Figure S3.** Locomotive activities analysis for monkeys re-entering experienced cages. Locomotive activities tracked from 20-minute video recordings. All mutant monkeys remained largely stationary when released to the same cage experienced one-month earlier, as revealed by the tracking marks. (A) Activity tracks (marked red) obtained by 20-minute videotape recordings. (B) Summary of locomotive activities, including total distance, moving velocity, stationary time, and (C) time spent on-ground *versus* time spent off-ground were analyzed. In black, blue and red indicated BMAL1-WT, BMAL1 mosaic, BMAL-KO monkeys, respectively. Data included two wild-type age-matched male monkeys, in addition to the 8 monkeys listed in Fig. 1B. ^🞱^*P* < 0.05; NS, not significant; Student’s *t*-test.


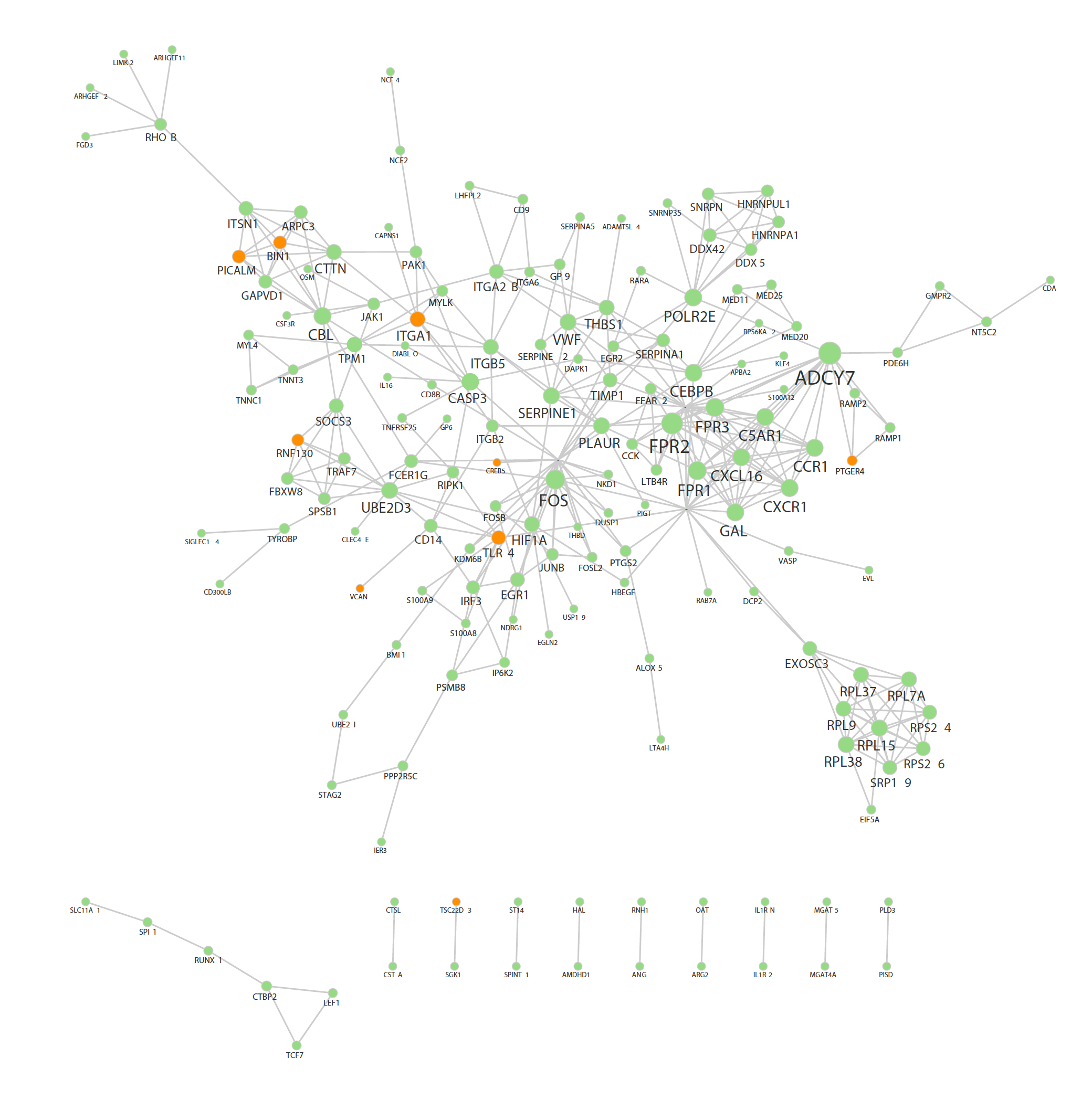


**Figure S4.** STRING network assembly of up-regulated targets in BMAL1- ablated A6. STRING network assembly of BMAL1-KO A6 monkey. Targets related to aging (GSE75337) were labeled in orange

**Table S1. Sequences of sgRNAs and summaries of the off-target analysis for BMAL1-edited cynomolgus monkey used in this study.**

Off-target (OT) sites that were analyzed for sgRNA1, sgRNA3, and sgRNA5. In lower case indicated mismatched nucleotides with compared to the respective sgRNA sequences. Sequences that were consistent to reference without error were labeled as “-”. Nucleotides in red indicated SNP occurred in the analysis, for which the SNPs were confirmed to originate from the parental sequences and labeled as “P”(paternal) or “M”(maternal).

**
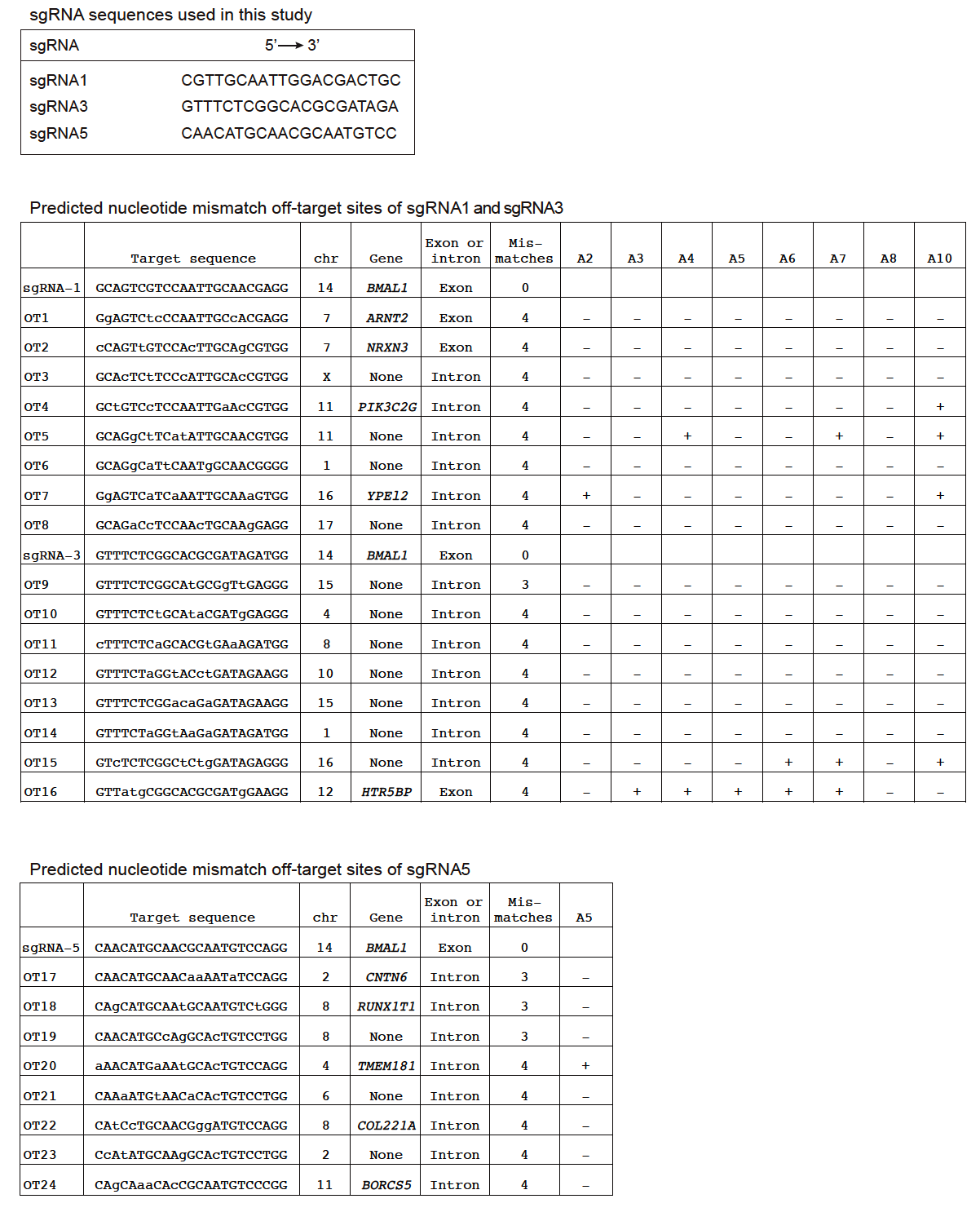
**

**Table S2. Sequences of primers used for qPCR and Off-target analysis in this study.**

**
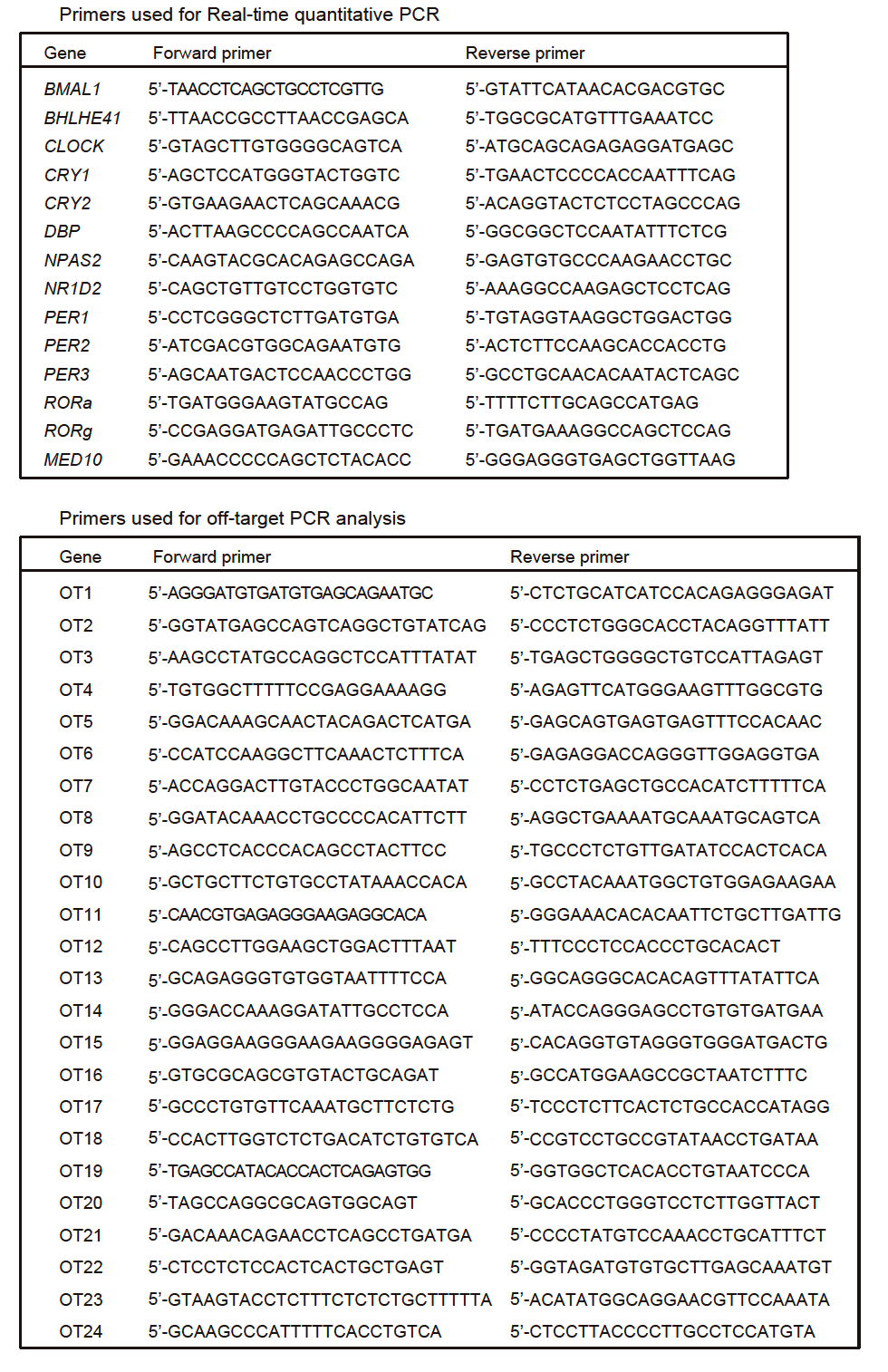
**

**
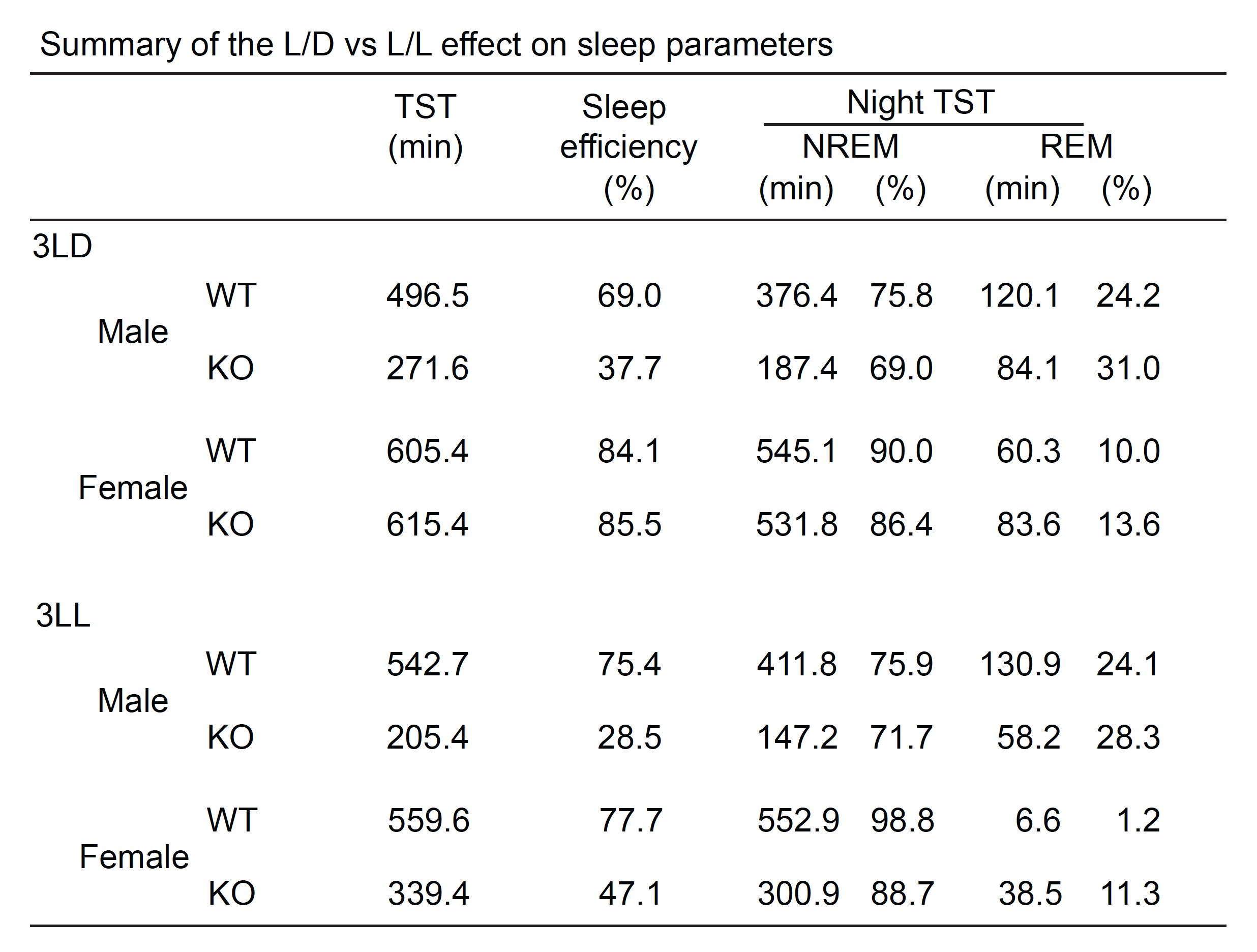
Table S3. Summary of total sleep time and sleep proportions associated to Fig. 3.**

**Table S4. List of transcripts with greater than 2-fold expressional changes in A5 versus A6.**

**Table S5. Significant GO terms with the list of greater than 2-fold changed transcripts in A5 versus A6.**

**Movie S1.**

20-minute video recording of BMAL1-WT A5 monkey used for behavioral analysis in Fig. 4, A-C. The file is formatted in 16X accelerated display for easier comparison with other monkeys.

**Movie S2.**

20-minute video recording of BMAL1-KO A6 monkey used for behavioral analysis in Fig. 4, A-C. The file is formatted in 16X accelerated display for easier comparison with other monkeys.

**Movie S3.**

20-minute video recording of BMAL1-WT A7 monkey used for behavioral analysis in Fig. 4, A-C. The file is formatted in 16X accelerated display for easier comparison with other monkeys.

**Movie S4.**

20-minute video recording of BMAL1-KO A3 monkey used for behavioral analysis in Fig. 4, A-C. The file is formatted in 16X accelerated display for easier comparison with other monkeys.

**Movie S5.**

15-second video recording of BMAL1-WT A5 monkey in the presence of care personnel.

**Movie S6.**

15-second video recording of BMAL1-KO A6 monkey in the presence of care personnel.
