# Supplementary material for: BMAL1 knockout macaque monkeys display reduced sleep and psychiatric disorders
Source: Natl Sci Rev. 2019 Jan 24;6(1):87–100. doi: 10.1093/nsr/nwz002 (PMC8291534; doi:10.1093/nsr/nwz002)
Supplement: Supplementary Files [file nwz002_supplemental_files.zip › NSR_MS-2018-233.R2.docx]

**RESEARCH ARTICLE**

**NEUROSCIENCE**

**BMAL1 knockout macaque monkeys display reduced sleep and psychiatric disorders**

Peiyuan Qiu^1,†^, Jian Jiang^1,3,†^, Zhen Liu^1,†^, Yijun Cai^1^, Tao Huang^2^, Yan Wang^1^, Qiming Liu^1^, Yanhong Nie^1^, Fang Liu^1,4^, Jiumu Cheng^1^, Qing Li^1^, Yun-Chi Tang^2^, Mu-ming Poo^1^, Qiang Sun^1^*, Hung-Chun Chang^1,3^*

^1^Institute of Neuroscience, State Key Laboratory of Neuroscience, CAS Key Laboratory of Primate Neurobiology, CAS Center for Excellence in Brain Science and Intelligence Technology, Chinese Academy of Sciences, Shanghai 200031, China.

^2^CAS Key Laboratory of Tissue Microenvironment and Tumor, Shanghai Institute of Nutrition and Health, Shanghai Institutes for Biological Sciences, University of Chinese Academy of Sciences, Shanghai 200031, China.

^3^Dynamic Brain Signal Analysis Facility, Institute of Neuroscience. Chinese Academy of Sciences, Shanghai 200031, China.

^4^College of Life Sciences, University of Chinese Academy of Sciences, Beijing 100049, China.

*Corresponding authors. E-mails: [hcchang@ion.ac.cn](mailto:hcchang@ion.ac.cn); [qsun@ion.ac.cn](mailto:qsun@ion.ac.cn).

^†^Equally contributed to this work.

**Abstract**

Circadian disruption is a risk factor for metabolic, psychiatric and age-related disorders, and non-human primate models could help to develop therapeutic treatments. Here we report the generation of BMAL1 knockout cynomolgus monkeys for circadian-related disorders by CRISPR/Cas9 editing of monkey embryos. These monkeys showed higher nocturnal locomotion and reduced sleep, which was further exacerbated by constant-light regimen. Physiological circadian disruption was reflected by the markedly dampened and arrhythmic blood hormonal levels. Furthermore, BMAL1-deficient monkeys exhibited anxiety and depression, consistent with their stably elevated blood cortisol, and defective sensory processing in auditory oddball tests found in schizophrenia patients. Ablation of BMAL1 up-regulated transcriptional programs toward inflammatory and stress responses, with transcription networks associated with human sleep deprivation, major depressive disorder, and aging. Thus, BMAL1 knockout monkeys are potentially useful for studying physiological consequences of circadian disturbance, and for developing therapies for circadian and psychiatric disorders.

**Keywords:** circadian rhythms, BMAL1, macaque monkey, sleep disruption, psychosis, aging

Received: 04-Dec-2018; Revised: 30-Dec-2018; Accepted: 31-Dec-2018.

**Introduction**

The circadian clock governs multiple physiological activities, including diurnal cycle, body temperature, metabolic rhythms, feeding, and sleep [1-3]. Experimental disruption of circadian rhythm in flies and rodents results in phenotypes resembling symptoms associated with numerous diseases, such as sleep disorders, cardiovascular dysfunction, diabetic mellitus, cancer, and neurodegenerative diseases [1, 4, 5]. Notably, all of these are chronic conditions highly correlated with aging progression [1, 4, 6, 7]. Many clinical studies have identified circadian gene mutations as vital causative factors for psychiatric disorders [8, 9]. However, due to limited phenotypes found in standard rodent models, particularly those related to psychiatric disorders, the molecular basis underlying the circadian-psychosis link remains unclear. To recapitulate clinical conditions associated with circadian rhythm dysfunction, diurnal animals with behavioral and metabolic properties closer to humans are highly desirable. Non-human primates thus represent the best choice for this purpose [10, 11]. With rapid advances in gene-editing technologies, genetic manipulation of non-human primates is now feasible [12, 13], and methods for shortening the long reproductive cycle of non-human primates were also developed [14]. Several transgenic and gene-edited non-human primates have been generated as potential animal models for metabolic stress, neurodegenerative diseases, immunodeficiency, autism, and perinatal lethality [12, 15-19].

Brain and Muscle ARNT-Like 1 (BMAL1) is a key component of CLOCK-BMAL1 transcription factor complex that activates the expression of a great majority of circadian genes. Deletion of BMAL1 in mice results in strong arrhythmic phenotypes, glucose intolerance, and premature aging [20-22]. In this study, we ablated BMAL1 in cynomolgus monkeys (*Macaca fascicularis*), and observed arrhythmic circadian activities in pre-adolescent monkeys, with altered Rapid Eye Movement (REM) and non-REM sleep, disrupted circadian cycling of many hormones, as well as behaviors resembling anxiety, depression, and schizophrenia in humans. Together with results from transcriptional network analysis, our findings provide a direct molecular link between circadian dysregulation and multiple diseases, and indicate the usefulness of non-human primates as animal models for studying pathogenic mechanisms underlying circadian-related disorders and for developing intervention strategies.

**Results**

***BMAL1* editing in cynomolgus monkeys**

To generate *BMAL1*-edited macaque monkeys, we first tested a battery of sgRNAs targeting *exon*s 8, 10 or 13 of the *BMAL1* locus in cultured embryonic monkey cell lines. We found that a combination of sgRNA1 and 3 (Fig. 1A and Table S1), which targeted *exon* 13 that encodes a crucial PAS domain for BMAL1 transcriptional activity, was the most efficient site for *BMAL1* editing. We then performed CRISPR/Cas9 editing of cynomolgus zygotes, which were obtained by intracytoplasmic sperm injection (ICSI), for targeting *BMAL1* *exon* 13 with sgRNA1 and 3. For comparison, we also edited a separate group of zygotes using sgRNA5 targeting *exon* 8. In total, 88 edited embryos were transferred to 31 surrogate recipient monkeys and 10 pregnancies were obtained, yielding 8 healthy live births and 2 spontaneously aborted fetuses (Fig. 1B). Among the 8 live animals, 5 showed mutated *BMAL1,* and 3 exhibited wild-type *BMAL1* (wild type, “WT”), as indicated by PCR analysis of skin samples. *BMAL1* was found to be mutated in all examined skin cells in female A3 and males A6, A8 (knockout, “KO”), with mutations involving base pair deletion (“-”), insertion (“+”), and point mutation (“pm”) (Fig. 1C). By contrast, A4 and A10 were partially edited for *BMAL1* (“mosaic”) (Fig. 1B-D). The subsequent whole-genome sequencing followed by off-target PCR analysis from blood samples demonstrated that *BMAL1*-editing was specific in this cohort (Table S1 and S2). Peripheral blood mRNA analysis confirmed the complete loss of the *BMAL1* transcript in BMAL1-KO monkeys A3, A6 and A8, resulting in down-regulation of several core clock genes as expected (Fig. 1E and Fig. S1). Immunoblot analysis on aborted fetuses A1 and A9 also revealed complete absence of BMAL1 and the consequent PER2 down-regulation in multiple tissues (Fig. 1F), consistent with the anticipated circadian disturbance at the molecular level.

**Abnormal nocturnal activity and blood hormones in BMAL1-KO monkeys**

To study the locomotor activity of *BMAL1*-edited monkeys, we tracked their voluntary locomotion with telemetric actimeters under 12-h light/12-h dark condition (“L/D”) for 14 days (see Methods). We found that BMAL1-KO monkeys displayed increased nocturnal active phenotype at 10 months after birth (Fig. 2A), particularly in the male monkey A6. As summarized for all 14 days (Fig. 2B), we observed higher overall nocturnal locomotor activities in both A6 and A3 monkeys, as compared to BMAL1-WT monkeys, with marked elevation of activities during late night in A6. Periodical analysis also revealed circadian rhythm irregularity, with BMAL1-KO animals showing multiple circadian periods (Fig. 2C). The results supported the conclusion that BMAL1 deficiency caused circadian locomotor abnormality, as that found in *Bmal1* KO mouse [20].

Daily cyclic release of neuroendocrine hormones is vital for supporting circadian-dependent downstream effector activities [23]. Dampened cyclic levels of hormones, particularly melatonin, were considered a cause of circadian dys-synchrony with aging [24]. Due to the strong association of circadian-related neuroendocrine factors with healthy physiology, we examined the rhythmic change of plasma melatonin level, which normally peaks at early night and drops to the lowest level during mid-day. By assaying blood melatonin levels every 6 hr over two days, we found that control monkeys (A2, A5, A7) showed normal rhythmic melatonin expression, but BMAL1-deficient monkeys, including A3, A6, and A8 (BMAL1-KO) as well as BMAL1 mosaic A4 and A10, all showed lower melatonin level without obvious rhythmicity (Fig. 2D and 2E), reminiscent of that found in aged rhesus monkeys [25]. The levels of testosterone and dehydroepiandrosterone (DHEA) were also higher and rhythmically expressed in control monkeys, but greatly dampened to low levels in BMAL1-deficient monkeys, again resembling aging-related disturbance of these hormones in rhesus monkeys [26]. Notably, we found that the usual cortisol decline at early night in WT monkeys was essentially absent in BMAL1-deficient monkeys (Fig. 2D and 2E). As shown later, this stably elevated cortisol level is consistent with the findings of behavioral phenotypes resembling psychiatric disorders [27].

**Disrupted sleep states and diurnal EEG oscillations in BMAL1-KO monkeys**

The disrupted melatonin level together with arrhythmic locomotor activity found in BMAL1-KO monkey implicated an imbalanced sleep homeostasis. Indeed, by analyzing the sleep/awake state-specific EEG power spectra, we found that monkey A6 under normal L/D condition exhibited higher awake state as well as lower rapid eye-movement (REM) and non-REM (NREM) states at night, as compared to those in control monkey A5. The reduced sleep time in monkey A6 is opposite to that found in *Bmal1* KO mice, implicating that major differences in BMAL1 regulation may exist between diurnal and nocturnal animal models [28]. These differences were even more pronounced for monkey A6 under 3-day constant light illumination (“L/L”), which was designed to examine the internal circadian maintenance in response to disturbed external cue (Fig. 3A and 3B). (Constant dark condition was not practical in our study due to animal care requirements). For the awake and sleep states, we did not find obvious difference between female monkey A3 and the control monkey A7 under the normal L/D condition, but clear disruption was observed in A3 under L/L condition, especially during the 2nd and 3rd day of L/L exposure (Fig. 3C and 3D). The awake/sleep EEG data were summarized by pie charts in Fig. 3 (B and D) and Table S3. Rhythmic changes of body temperature were often used as a functional index of circadian rhythm. We found that fluctuations in body temperature in BMAL1-KO monkeys were much larger than those in control monkeys, particularly under the L/L condition. The daily cycle of body temperature fluctuations persisted in control monkeys, but was essentially abolished in both BMAL1-KO monkeys on day 2 and 3 after switching to the L/L condition (Fig. S2).

**Psychosis-like behavioral phenotypes in BMAL1-deficient monkeys**

The constant high-level cortisol implicates a potential predisposition to stress and depression [29]. Indeed, videotape behavior tracking of BMAL1-deficient monkeys in the novel environment (of a new cage) revealed several abnormalities. First, in contrast to the active exploration of control monkeys, BMAL1-deficient monkeys remained largely stationary over the 20-min recording period, indicative of a stressed condition (Fig. 4A and 4B) [30]. Furthermore, the area covered by locomotor activities was restricted to corners away from the corridor, and the time spent off-ground were significantly higher (Fig. 4C), reminiscent of the reduced cage exploration of monkeys due to stress [31]. These phenotypes were most pronounced in BMAL1-KO monkeys A6 and A3, as illustrated in Fig. 4C (see Movie S1- S4). The monkey A6 exhibited particularly strong fear and anxiety, showing clear avoidance to the care personnel in his home cage by retreating to a cage corner, and buried the head with hands (Movie S5 and S6). Notably, the latter behaviors were not observed when A6 was undisturbed, suggesting that this abnormality was triggered by external inputs. When these BMAL1-deficient monkeys were re-exposed to the same cage one-month later, we observed similar phenotypes of higher tendency in remaining stationary and staying off-ground when moved, as compared to control monkeys (Fig. S3). These behaviors are consistent with neuroendocrine dysfunctions, as exemplified by the persistently elevated cortisol level that is known to be associated with depression and anxiety [29, 32].

Using auditory oddball test, which measures perception of infrequent changes in normal sound sequences, as reflected by mismatch negativity (MMN) response in event-related potential (ERP)[33], we found that BMAL1-KO monkeys A6 and A3 were defective in MMN responses. The average peak MMN amplitudes after the deviant stimulus onset (range 100 to 150 ms) were significantly lower than that found in BMAL1-WT A5 and A7, as shown by the example heat maps of ERP amplitudes over the entire scalp mapped with 21 EEG electrodes (Fig. 4D). Roster plots of ERP amplitude for 60 deviant trials recorded by Fz electrode (marked by red ^🞱^ in Fig. 4D) showed reduced repeatability of MMN in ERPs induced by deviant stimuli in BMAL1-KO monkeys (Fig. 4E), as also presented by the summary plots of ERPs during 240 regular vs. 60 deviant trials (Fig. 4F). The average ERPs of control and BMAL1-KO monkeys evoked by regular and deviant sounds (Fig. 4G) and the difference of regular vs. deviant ERPs for two types of monkeys (Fig. 4H) both showed marked reduction in MMN amplitudes for the BMAL1-KO monkeys. The impaired cognitive function in novelty detection suggests BMAL1 deficiency had triggered a schizophrenia-like symptom [34]. Taken together, the behavioral and electrophysiological results indicated that BMAL1-KO monkeys represent a potential non-human primate model for studying the causal link between circadian disorders and psychosis.

**Transcriptome analysis revealed inflammatory dysregulation and chronic maladies**

To examine the impact of BMAL1 ablation on transcriptional activities, we carried out a temporal analysis of blood samples from control monkey A5 and BMAL1-KO monkey A6 for RNA sequencing at different time points (ZT2, 8, 14 and 20). As expected, we found altered expression of a large number of circadian-controlled transcripts in A6 samples, mostly showing reduced expression (Fig. 5A). Among core circadian genes, *RORβ,* *PER1* and *NPAS2* also exhibited the absence of circadian phasic expression (Fig. 5A). Among 1402 transcripts identified with 2-fold expressional changes between A5 and A6 (Fig. 5B, 5C and Table S4), we noticed a group of inflammatory targets that were elevated and dysregulated throughout all four time points, including *IL1R2* and *NFKBIZ*, suggesting systemic inflammation was induced by circadian disruption (Fig. 5B, 5C and Table S4). Gene ontology (GO) analysis of up-regulated genes also showed that immune system processes and inflammatory responses were high on the list (Fig. 5C). This is consistent with the findings that the immune system is rhythmically regulated along with the circadian program for its activation and suppression homeostasis [35], and that BMAL1 plays a suppressive role in inflammatory monocyte populations [36]. Other biological processes revealed in GO terms included stress responses, response to stimulus, and locomotion (Fig. 5C and Table S5), correlating well with psychiatric phenotypes described above for BMAL1-KO monkey A6.

Transcriptome assembly depicted by STRING indicated a distinct functional network activated by BMAL1 ablation. Re-assigned program with up-regulated clusters such as *BRD2*, *DPP4*, *DDX5* implicate inflammatory, metabolic imbalance, and predisposed oncogenic risks, respectively (Fig. 5D and 5E). To further explore the potential dysfunctions connected to circadian disorders due to BMAL1 deficiency, we compared blood transcriptome databases for human subjects with sleep deprivation (GSE39445) [37], major depressive disorder (GSE76826) [38], and aging (GSE75337) [39]. We found significant correlations between up-regulated genes in A6 and those associated with the above three human conditions. Many genes, e.g., *FOS*, *DUSP1,* and *HNRNPA1*, were up-regulated in more than one conditions, suggesting potential molecular links between sleep deprivation and mood disorders (Fig. 5F). Perhaps most interesting genes are those up-regulated in all three conditions, such as *TLR4* and *CREB5*, which may serve as vital triggers for pathways that link circadian disorders to chronic diseases and aging (Fig. 5F and Fig. S4). Together, these results suggest that the macaque model described here may be useful for probing cellular and molecular mechanisms underlying circadian-related disorders.

**Discussion**

The progress in translating basic research findings to treatments of human diseases was often limited by the availability of animal models that mimic symptoms of human disorders. A suitable model for translational purposes should be complex enough to display as many phenotypes as those observed in humans, and exhibit physiological and anatomical features close to human. For example, the intrinsic difference in diurnal versus nocturnal activity patterns may blur the effects of external perturbation, e.g., light/dark cycle on molecular oscillations and interfere with the design of therapeutic treatments. A recent diurnal transcriptome atlas derived from baboon experiments indicated distinct phases of rhythmic gene expression from those found in mice [40], suggesting the need for non-human primate models beyond nocturnal rodents. The present BMAL1-deficient monkey is a preferred model for circadian disruption for the following reasons. First, BMAL1 exerts crucial activity in transcriptionally regulating circadian program in most tissues, thus the ablation offered opportunities in investigating both central and peripheral clocks with minimal concern of genetic redundancy. Second, BMAL1 knockout in mice caused circadian arrhythmicity, sleep and metabolic disorders [4, 41], as well as premature aging [22, 42], a non-human primate model could be useful for multiple disease areas in supplement of deficiencies found in *Bmal1* mice. Finally, BMAL1 has been linked to psychiatric conditions [8, 9]. Studies on the effects of BMAL1 ablation in monkey model, together with appropriate behavioral assays, could help us to understand the molecular basis of the circadian link to mood disorders.

Useful rodent models have been developed for studying circadian dysfunction and mood disorders [43], including suprachiasmatic nucleus-disruption [44] and *Clock-Δ19* and *Per1^Brdm1-/-^* mutations [45, 46]. The present monkey model here offered additional observations on behaviors relating to psychiatric disorders. Stressed behaviors in BMAL1-KO monkeys, such as having the head buried in hands (Movie S6), avoiding care personnel, and schizophrenia-related cognitive impairment in auditory oddball MMN trials, were rather unique among animal models. Further interventional studies aiming at ameliorating specific behavioral abnormalities in BMAL1-deficient monkeys will be useful for developing psychiatric treatments.

The transcriptome results indicated over-representation of immunological and inflammatory responses upon BMAL1 ablation, consistent with the findings that several immune mediators are affected by circadian oscillations, thus weakening clock programs can lead to immune dysregulation [47], including abnormal regulation of toll-like-receptor 4/TNFα pathway in myeloid cells [48] and the activation of inflammatory monocytes [35, 36]. Longitudinal analyses of BMAL1-KO monkeys will help to understand the pathogenesis and develop the therapeutics for inflammatory chronic diseases induced by circadian dysfunctions.

METHODS

**Animal ethics statement**

The use and care of cynomolgus monkeys (*Macaca fascicularis*) complied with the guideline of the Animal Advisory Committee at the Shanghai Institutes for Biological Science, Chinese Academy of Sciences (CAS), under the approval application entitled ‘Reproductive physiology of cynomolgus monkeys and establishment transgenic monkeys’(ER-SIBS-221106P). The monkeys in the process of experiment were housed in a conditioned environment (temperature: 22 ± 1^0^C; humidity: 50% ±5% RH) with 12 hr light/12 hr dark cycle (light on time 07:00 to 19:00). All animals were fed with commercial monkey diet (Anmufei, Suzhou) twice a day with free access tap water, and with fruits and vegetables supplements once daily. Animals have been under careful veterinary surveillance to ensure health conditions during and after experiments.

**Superovulation, oocyte collection and gene editing**

Healthy female cynomolgus monkeys with regular menstrual cycles were chosen for superovulation, and oocyte collections were carried out via laparoscopy. From day 3 of the menstrual cycle, 25 IU recombinant human follitropin was injected intramuscularly twice daily for 7–8 days. On day 11, 1000 IU of human chorionic gonadotrophin (hCG) was administrated, followed by oocyte collection from follicles (2-8 mm in diameter) 36 h later. The collected oocytes were cultured in the pre-equilibrated hamster embryo culture medium 9 (HECM-9) medium. Metaphase II-arrested oocytes were selected for further manipulations [49]. *Cas9* and sgRNA mRNA were prepared as previously reported [50]. For sgRNA preparation, a T7 promoter contained specific forward and a common reverse primers were used to amplify the sgRNA template by PCR, and the resulted PCR product was used for in vitro transcription by using MEGAshortscript T7 kit (Thermo Fisher Scientific). For *Cas9* mRNA, a T7 promoter contained specific Forward and a common Reverse primer were used to amplify the *Cas9* coding region and the resulted PCR product was used for in vitro transcription by using mMESSAGE mMACHINE T7 ULTRA kit (Thermo Fisher Scientific). *Cas9* mRNA and sgRNA purifications were achieved by using MEGAclear kit.

Fertilization was performed via intra-cytoplasmic sperm injection (ICSI) [49], and confirmed for the presence of two pronuclei and two polar bodies. CRISPR/Cas9 method was applied for *BMAL1* (Gene ID 101865448) gene editing in the zygotes. The design of sgRNA followed the instruction of website (http://crispr.mit.edu/job/9950152458235442), and the sequences are listed in Figure 1A and Table S1. Approximately 5 pl of 50 ng/μl sgRNAs and 100 ng/μl in vitro transcribed *Cas9* mRNA were mixed and injected in the cytoplasm of fertilized oocytes. Injected embryos were cultured in HECM-9 media containing 5% fetal bovine serum at 37^0^C in 5% CO_2_ to allow embryo development. 31 menstrual synchronized females were used as surrogate recipients for sgRNA-*Cas9* injected embryos. Typically, 2 pronuclear-early stage embryos were selected for tubal transfer to each surrogate female [49].

**Genotyping Analysis**

Tissues including ear skin fibroblasts, peripheral blood from founder monkeys were collected and digested in lysis buffer (10 mM Tris-HCl, 0.4 M NaCl, 2 mM EDTA, 1% SDS and 100 mg/ml Proteinase K) overnight at 65 °C. The genomic DNA was then purified by phenol-chloroform extraction and alcohol precipitation. PCR was performed using targeted gene-specific primers

*BMAL1*-F: 5’ TGGTGTATGTAATGAATGCCCATGG 3’;

*BMAL1*-R: 5’ TAGAGACAGGGTTTTGCCATGTTGC 3’ and PCR products were then sub-cloned to pMD19-T vector for sequencing and verifying the mutations as showed in Fig. 1C.

**Off-target analysis**

The genomes of CRISPR/Cas9 edited monkeys were sequenced using Illumina NovaSeq 6000 System (Illumina, San Diego, CA, USA). Qualified reads were mapped to the assembly *Macaca fascicularis* genome (v5) using BWA (v0.7.12-r1044) and the variants/indels were identified using Sentieon Genomics (ver.201611.02). The potential off-target sites were predicted using a genome-wide CRISPR/Cas9 off-target site prediction tool Cas-OFFinder (<http://www.rgenome.net/cas-offinder/>) [51]. We first predicted the off-target sites with conditions that allowed 3-4 mismatches occurred in the sgRNA sequences of sgRNA1, 3, and 5. We further searched for SNPs/indels that occurred in the 5bp-spanning range at the predicted sequences. Cases with SNPs/indels occurred in the analysis were further subjected to PCR and Sanger sequencing validation of using blood DNA samples, together with respective parental PCR-sequencing assays to ascertain the source of sequence variations. The off-target analysis results, and related primer information were summarized in Table S1 and S2, respectively.

**Immunoblot**

Brain, heart, kidney, liver, lung, muscle, and spleen tissues were carefully removed from stillbirth carcasses of A1 and A9 and stored at -80°C until use. Approximately 50 mg tissue samples were homogenized and lysed in ice-cold lysis buffer (50 mM Tris at pH 7.5, 150 mM NaCl, 1% Triton X-100, 0.5% NP-40 and 10% glycerol) supplemented with complete protease inhibitor (Roche). Protein levels were standardized using the Bradford protein assay before boiled in Laemmli buffer. Protein samples (25 μg) were resolved in 10% acrylamide gel then transferred to PVDF membrane, followed by standard protocols for immunoblotting with the following primary antibodies: rabbit anti-BMAL1 (CST14020S, Cell Signaling Technology), rabbit anti-PER2 (AB2202, Merck Millipore), mouse anti-GAPDH (60004-1-1g, Proteintech); and secondary antibodies: Goat anti-rabbit IgG (H + L)-HRP conjugate (1706515, Bio-Rad), Goat anti-mouse IgG (H + L)-HRP conjugate (1706516, Bio-Rad). Consistent results were obtained from three independent immunoblot sets.

**Blood sample collection**

The BMAL1 monkey cohort was studied for blood circadian transcript levels at 12- month of age. Blood hormonal levels and transcriptome analysis were inspected at 12-month of age. Blood samples (0.5 ml) were collected intravenously at time points ZT2 (9 am, 2 hours post light on), ZT8, ZT14 and ZT20 for transcriptional analyses, or same time points for 2-day continuous sampling for plasma assays. Briefly, blood samples for real-time quantitative PCR or transcriptome assay were pre-incubated with red cell lysis buffer (TIANGEN, RT122-02) to remove erythrocytes, followed by centrifugation at 1,200 × g at 4°C for 5 min. Leucocyte pellets were then flash-frozen in liquid nitrogen and stored at −80°C until the use for total RNA extraction. Blood samples for plasma assays were first collected into EDTA-coated Improvacuter^®^ vacuum blood collection tubes. The plasma fraction was obtained by centrifugation at 1,000 × g for 10 min at room temperature, then stored at -80°C until assay.

**Real-time quantitative PCR**

Total RNAs from leucocytes were first extracted using RNeasy Mini Kit (Qiagen), followed by cDNA reverse transcription with Omniscript RT Kit (Qiagen). Real-time PCR reactions were prepared with the use of QuantiNova SYBR Green PCR Kit (Qiagen), then analyzed on StepOnePlus Real-Time PCR system (ThermoFisher). The relative abundance of transcripts was calculated by normalizing to *Med10* level. Six monkeys were analyzed for circadian gene expressions including three age-matched wild-type monkeys; and the three BMAL1-KOs, A3, A6 and A8. Primers are listed in Table S2.

**Locomotor activity monitoring**

Locomotor activity was recorded in monkeys at near 10-month of age for 14 days in singly housed condition. Physical activity level was detected with Xenon ACT II wireless accelerometer (BLEACT; Cloud Care Technologies) that was designed to be light-weighted (7 g) and small (4 cm * 1.5 cm * 1.1 cm) for easy, long term wearing purpose [52]. Individual accelerometer was secured in a plastic collar thus facilitated continuous wearing for the monkeys. The locomotion activity signals were analyzed with ClockLab Analysis software (Actimetrics, Version 6.0) for the averaged activity amplitudes over 14 days of recording, and the free-running chi-squared periodogram under standard12 hr light/12 hr dark cycle.

**Plasma ELISA assays**

Melatonin, testosterone, DHEA, and cortisol levels were assayed via ELISA kits according to the manufacturer’s protocols (BioSource MBS743125, Enzo ADI-900-065, Enzo ADI-900-093 and Enzo ADI-900-071, respectively). Briefly, plasma samples collected from time points were first aliquoted for either applied directly in the melatonin assay, or extracted with equal volume of ethyl acetate (DHEA), or diethyl ether (for testosterone and cortisol) for 3 times. The extracted materials were lyophilized then reconstituted in respective assay buffers provided in the kits, followed by assaying in a 96-well format plate with incubation, wash and colorimetric measurement steps as described in the manufacturer’s protocols.

**Sleep recording and EEG analysis**

Wake-sleep activities were analyzed in monkeys at near 15-month of age. Sleep recording was carried out by chronically implanting radio-telemeter transmitters (PhysioTel Digital M01 implant, Data Sciences International) for continuous long-term measuring of electroencephalography (EEG) and electromyography (EMG) signals. Monkeys were deeply-anesthetized by giving intramuscularly dosing of 5 mg/kg Zoletil 50 (Virbac S.A.) before implanting surgery. In case the surgery exceeds 2 hours, supplement of additional 1 mg/kg Zoletil 50 was provided intramuscularly to maintain deep anesthetization. The DSI implant was embedded subcutaneously in the shoulder-back (at the subscapular region). The EEG electrode bio-potential leads were subcutaneously tunneled to the skull, and the two EEG electrodes were screwed into the skull 5 mm lateral, and 5 or 10 mm anterior, respectively, to the lambda midline. Electrode leads for EMG was sutured to the neck musculature. After surgery, monkeys were returned to their home cage with analgesia Ketoprofen (1 mg/kg) provided via intramuscularly dosing daily for 3 days, and antibiotics (Penicillin, 20 MU/kg) provided daily for 1 week. The room for EEG/EMG recording (5.5 × 2.5 × 2.9 m) was equipped with 6 transceivers (TRX-1) mounted to the walls thus allowed excellent sampling of telemetric signals transmitted from anywhere in the room. The TRX-1 transceivers were connected to a data exchange matrix (CLC) then to a computer for recording and data storage. Sleep recording was performed for 3 days under normal illuminating condition, i.e., light on time 07:00 to 19:00, and then switched to recording under full day lighting for another 3 days. Wake–sleep stage scoring was performed with software NeuroScore 3.2.1 (DSI) for identifying stages as wake, rapid eye movement (REM) sleep, and slow-wave non-REM (NREM) sleep. The results were depicted every 60 min for the stage proportions for the 3-day period, as showed in Figure 2. Briefly, EEG signals sampled at 448 Hz were ﬁltered and analyzed by time frequency fast-Fourier transform (FFT) analysis with a 10-s epoch Hanning window. Gamma (24–100 Hz), beta (16–24 Hz), sigma (12-16 Hz), alpha (8–12 Hz), theta (4–8 Hz), and delta (0.5–4 Hz) frequencies were applied as default NeuroScore 3.2.1 settings for wake-sleep stage scorings.

**Locomotor behavior observation and analysis**

Monkey locomotion behaviors were monitored at the age of 16 months, with video-recording during the light cycle in an observation cage (1.5 × 1 × 1.1 m) for 20 min without interruption. The new cage exploring videos were scored using the EthoVision XT software Version 11.5 (Noldus Information Technology). Locomotor activities after tracking were categorized for the results of moving distance, moving velocity and stationary time. The off-ground activity was calculated for the time when all limbs leave the ground.

**Scalp EEG recording and analysis for auditory-intensity oddball paradigm**

The passive auditory-intensity oddball paradigm was designed to present 100 ms tones in different intensities (low, 60 dB; high, 100 dB) to monkey subjects. Stimuli of frequent (standard, 240 events, 80% in the recording) and infrequent (deviant, 60 events, 20% in the recording) intensities were presented, with an inter-stimulus interval setting of 700 ms. 100 dB high-deviant condition was applied in the study. Stimulus presentation was controlled by E-prime 3.0 using a personal computer. Tones were presented using a HP amplifier about 10 centimeters away from subject ears. Ag/AgCl electrode, 22-channel EEG cap was applied for scalp EEG recordings, and activity signals were acquired with Neuroscan SynAmps RT amplifier and the Curry 8 software (Neuroscan). EEG data were then analyzed using MATLAB, with data pre-processing procedures including 50Hz notch filtering, dataset referencing, band-pass filtering, and segmentation, prior to event-related potential (ERP) calculation. The topographic voltage-distribution maps were generated with coordinating all electrodes from three-dimensional positions to a two-dimensional projection by MATLAB. The maps for average potential value reflecting MMN (100-150 ms) were shown.

**RNA-Sequencing and Data Analysis**

Peripheral blood samples were collected at ZT2, ZT8, ZT14, ZT20 from BMAL1 monkeys A5 and A6, and total RNAs from leucocytes were extracted using RNeasy Mini Kit (Qiagen). The RNA samples were examined with Agilent 2100 Bioanalyzer to ensure RNA integrity number (RIN) > 8.0, before subjected to cDNA library construction and further RNAseq analysis. Libraries were prepared using Illumina TruSeq RNA Library Prep Kit v2, then sequenced via Illumina Hiseq platform at 150 bp paired-end reads generated. The sequencing data were mapped to monkey genome (NCBI: *Macaca_fascicularis*_5.0) using bowtie 2 software. Gene expression levels were quantified as FPKM (fragments per kilobase of exon per million mapped fragments) using Cufflinks [53]. For differentially expressed genes between A5 and A6 samples with fold change greater than 2, we plotted the gene expression heatmap, performed Gene Ontology (GO) enrichment analysis and constructed the functional association network based on STRING database (version 10.5). The blood gene expression signatures for sleep deprivation (GSE39445), major depressive disorder (GSE76826) and aging (GSE75337) derived from Gene Expression Omnibus (GEO) database were applied to annotate the up-regulated transcripts in BMAL1-KO A6.

**Statistical analyses**

We used Student’s *t-*test for the following comparisons: for pairwise comparisons of new cage exploration experiments as shown in Fig. 4A-C, and Fig. S3A-C; for the mean potential values induced by two different tone intensities (regular vs deviant) with time in Fig 4F-H. Statistical tests were conducted using Prism (GraphPad) or MATLAB.

**Data availability**

Data generated during this study are available in the Sequence Read Archive (SRA) repository under accession numbers SRP145518 for whole genome sequence, and SRP145029 for RNA-seq raw data of cynomolgus monkeys. All codes are available upon request.

**Supplementary Data**

Figure S1-S4

Table S1-S5

Movie S1-S6

**Acknowledgments**

We thank T.B.J. Kuo and C.-H. Wu of National Yang-Ming University for technical support on the accelerometer BLEACT. Y. Lu, C. Zhang, L. Wang for animal care and transfer.

**Funding**

This work was supported by grants from the Strategic Priority Research Program of Chinese Academy of Science [XDB32060200 to H-C.C., XDB32060100 to Q.S. and XDB32070100 to M-m.P.], Shanghai Municipal Science and Technology Major Project [2018SHZDZX05 to H-C.C., Q.S. and M-m. P.], Shanghai Municipal Government Bureau of Science and Technology [18JC1410100 to H-C.C., Q.S. and M.-m. P.], Ministry of Science and Technology (973 Program, 2011CBA00400), Hundreds of Talents Program of CAS to H-C.C., National Natural Science Foundation of China [31671221 to H-C.C], Chinese Academy of Sciences Grant [QYZDY-SSW-SMC001 to M-m.P.], CAS Key Technology Talent Program to Q.S., and National Postdoctoral Program for Innovative Talents to Z.L..

**References**

1. Bass, J, Takahashi, JS. Circadian integration of metabolism and energetics. *Science*. 2010; **330**(6009): 1349-54.

2. Asher, G, Sassone-Corsi, P. Time for food: the intimate interplay between nutrition, metabolism, and the circadian clock. *Cell*. 2015; **161**(1): 84-92.

3. Allada, R, Cirelli, C, Sehgal, A. Molecular Mechanisms of Sleep Homeostasis in Flies and Mammals. *Cold Spring Harbor perspectives in biology*. 2017; **9**(8).

4. Mattis, J, Sehgal, A. Circadian Rhythms, Sleep, and Disorders of Aging. *Trends in endocrinology and metabolism: TEM*. 2016; **27**(4): 192-203.

5. Musiek, ES, Holtzman, DM. Mechanisms linking circadian clocks, sleep, and neurodegeneration. *Science*. 2016; **354**(6315): 1004-8.

6. Chang, HC, Guarente, L. SIRT1 mediates central circadian control in the SCN by a mechanism that decays with aging. *Cell*. 2013; **153**(7): 1448-60.

7. Liu, F, Chang, HC. Physiological links of circadian clock and biological clock of aging. *Protein & cell*. 2017; **8**(7): 477-88.

8. Lamont, EW, Legault-Coutu, D, Cermakian, N*, et al.* The role of circadian clock genes in mental disorders. *Dialogues in clinical neuroscience*. 2007; **9**(3): 333-42.

9. Charrier, A, Olliac, B, Roubertoux, P*, et al.* Clock Genes and Altered Sleep-Wake Rhythms: Their Role in the Development of Psychiatric Disorders. *International journal of molecular sciences*. 2017; **18**(5).

10. Izpisua Belmonte, JC, Callaway, EM, Caddick, SJ*, et al.* Brains, genes, and primates. *Neuron*. 2015; **86**(3): 617-31.

11. Jennings, CG, Landman, R, Zhou, Y*, et al.* Opportunities and challenges in modeling human brain disorders in transgenic primates. *Nature neuroscience*. 2016; **19**(9): 1123-30.

12. Niu, Y, Shen, B, Cui, Y*, et al.* Generation of gene-modified cynomolgus monkey via Cas9/RNA-mediated gene targeting in one-cell embryos. *Cell*. 2014; **156**(4): 836-43.

13. Zuo, E, Cai, YJ, Li, K*, et al.* One-step generation of complete gene knockout mice and monkeys by CRISPR/Cas9-mediated gene editing with multiple sgRNAs. *Cell Res*. 2017; **27**(7): 933-45.

14. Liu, Z, Nie, YH, Zhang, CC*, et al.* Generation of macaques with sperm derived from juvenile monkey testicular xenografts. *Cell Res*. 2016; **26**(1): 139-42.

15. Yang, SH, Cheng, PH, Banta, H*, et al.* Towards a transgenic model of Huntington's disease in a non-human primate. *Nature*. 2008; **453**(7197): 921-4.

16. Sato, K, Oiwa, R, Kumita, W*, et al.* Generation of a Nonhuman Primate Model of Severe Combined Immunodeficiency Using Highly Efficient Genome Editing. *Cell Stem Cell*. 2016; **19**(1): 127-38.

17. Chen, Y, Yu, J, Niu, Y*, et al.* Modeling Rett Syndrome Using TALEN-Edited MECP2 Mutant Cynomolgus Monkeys. *Cell*. 2017; **169**(5): 945-55 e10.

18. Liu, Z, Li, X, Zhang, JT*, et al.* Autism-like behaviours and germline transmission in transgenic monkeys overexpressing MeCP2. *Nature*. 2016; **530**(7588): 98-102.

19. Zhang, W, Wan, H, Feng, G*, et al.* SIRT6 deficiency results in developmental retardation in cynomolgus monkeys. *Nature*. 2018; **560**(7720): 661-5.

20. Bunger, MK, Wilsbacher, LD, Moran, SM*, et al.* Mop3 is an essential component of the master circadian pacemaker in mammals. *Cell*. 2000; **103**(7): 1009-17.

21. Marcheva, B, Ramsey, KM, Buhr, ED*, et al.* Disruption of the clock components CLOCK and BMAL1 leads to hypoinsulinaemia and diabetes. *Nature*. 2010; **466**(7306): 627-31.

22. Kondratov, RV, Kondratova, AA, Gorbacheva, VY*, et al.* Early aging and age-related pathologies in mice deficient in BMAL1, the core componentof the circadian clock. *Genes & development*. 2006; **20**(14): 1868-73.

23. Gamble, KL, Berry, R, Frank, SJ*, et al.* Circadian clock control of endocrine factors. *Nat Rev Endocrinol*. 2014; **10**(8): 466-75.

24. Reiter, RJ. The pineal gland and melatonin in relation to aging: a summary of the theories and of the data. *Experimental gerontology*. 1995; **30**(3-4): 199-212.

25. Urbanski, HF, Sorwell, KG. Age-related changes in neuroendocrine rhythmic function in the rhesus macaque. *Age*. 2012; **34**(5): 1111-21.

26. Downs, JL, Mattison, JA, Ingram, DK*, et al.* Effect of age and caloric restriction on circadian adrenal steroid rhythms in rhesus macaques. *Neurobiology of aging*. 2008; **29**(9): 1412-22.

27. Joseph, JJ, Golden, SH. Cortisol dysregulation: the bidirectional link between stress, depression, and type 2 diabetes mellitus. *Annals of the New York Academy of Sciences*. 2017; **1391**(1): 20-34.

28. Laposky, A, Easton, A, Dugovic, C*, et al.* Deletion of the mammalian circadian clock gene BMAL1/Mop3 alters baseline sleep architecture and the response to sleep deprivation. *Sleep*. 2005; **28**(4): 395-409.

29. Carroll, BJ, Cassidy, F, Naftolowitz, D*, et al.* Pathophysiology of hypercortisolism in depression. *Acta psychiatrica Scandinavica Supplementum*. 2007(433): 90-103.

30. Bornstein, SR, Engeland, WC, Ehrhart-Bornstein, M*, et al.* Dissociation of ACTH and glucocorticoids. *Trends in endocrinology and metabolism: TEM*. 2008; **19**(5): 175-80.

31. Camus, SM, Blois-Heulin, C, Li, Q*, et al.* Behavioural profiles in captive-bred cynomolgus macaques: towards monkey models of mental disorders? *PLoS One*. 2013; **8**(4): e62141.

32. Parker, KJ, Schatzberg, AF, Lyons, DM. Neuroendocrine aspects of hypercortisolism in major depression. *Hormones and behavior*. 2003; **43**(1): 60-6.

33. Gil-da-Costa, R, Stoner, GR, Fung, R*, et al.* Nonhuman primate model of schizophrenia using a noninvasive EEG method. *Proc Natl Acad Sci U S A*. 2013; **110**(38): 15425-30.

34. Featherstone, RE, Melnychenko, O, Siegel, SJ. Mismatch negativity in preclinical models of schizophrenia. *Schizophrenia research*. 2018; **191**: 35-42.

35. Man, K, Loudon, A, Chawla, A. Immunity around the clock. *Science*. 2016; **354**(6315): 999-1003.

36. Nguyen, KD, Fentress, SJ, Qiu, Y*, et al.* Circadian gene Bmal1 regulates diurnal oscillations of Ly6C(hi) inflammatory monocytes. *Science*. 2013; **341**(6153): 1483-8.

37. Moller-Levet, CS, Archer, SN, Bucca, G*, et al.* Effects of insufficient sleep on circadian rhythmicity and expression amplitude of the human blood transcriptome. *Proc Natl Acad Sci U S A*. 2013; **110**(12): E1132-41.

38. Miyata, S, Kurachi, M, Okano, Y*, et al.* Blood Transcriptomic Markers in Patients with Late-Onset Major Depressive Disorder. *PLoS One*. 2016; **11**(2): e0150262.

39. Aramillo Irizar, P, Schauble, S, Esser, D*, et al.* Transcriptomic alterations during ageing reflect the shift from cancer to degenerative diseases in the elderly. *Nat Commun*. 2018; **9**(1): 327.

40. Mure, LS, Le, HD, Benegiamo, G*, et al.* Diurnal transcriptome atlas of a primate across major neural and peripheral tissues. *Science*. 2018.

41. Lowrey, PL, Takahashi, JS. Genetics of circadian rhythms in Mammalian model organisms. *Advances in genetics*. 2011; **74**: 175-230.

42. Kondratova, AA, Kondratov, RV. The circadian clock and pathology of the ageing brain. *Nature reviews Neuroscience*. 2012; **13**(5): 325-35.

43. Menet, JS, Rosbash, M. When brain clocks lose track of time: cause or consequence of neuropsychiatric disorders. *Current opinion in neurobiology*. 2011; **21**(6): 849-57.

44. Landgraf, D, Long, JE, Proulx, CD*, et al.* Genetic Disruption of Circadian Rhythms in the Suprachiasmatic Nucleus Causes Helplessness, Behavioral Despair, and Anxiety-like Behavior in Mice. *Biological psychiatry*. 2016; **80**(11): 827-35.

45. Landgraf, D, McCarthy, MJ, Welsh, DK. Circadian clock and stress interactions in the molecular biology of psychiatric disorders. *Current psychiatry reports*. 2014; **16**(10): 483.

46. Roybal, K, Theobold, D, Graham, A*, et al.* Mania-like behavior induced by disruption of CLOCK. *Proc Natl Acad Sci U S A*. 2007; **104**(15): 6406-11.

47. Scheiermann, C, Gibbs, J, Ince, L*, et al.* Clocking in to immunity. *Nature reviews Immunology*. 2018; **18**(7): 423-37.

48. Keller, M, Mazuch, J, Abraham, U*, et al.* A circadian clock in macrophages controls inflammatory immune responses. *Proc Natl Acad Sci U S A*. 2009; **106**(50): 21407-12.

49. Liu, Z, Cai, Y, Sun, Q. Genome Editing of Monkey. *Methods Mol Biol*. 2017; **1630**: 141-52.

50. Wang, H, Yang, H, Shivalila, CS*, et al.* One-step generation of mice carrying mutations in multiple genes by CRISPR/Cas-mediated genome engineering. *Cell*. 2013; **153**(4): 910-8.

51. Bae, S, Park, J, Kim, JS. Cas-OFFinder: a fast and versatile algorithm that searches for potential off-target sites of Cas9 RNA-guided endonucleases. *Bioinformatics*. 2014; **30**(10): 1473-5.

52. Kuo, TBJ, Li, JY, Chen, CY*, et al.* Influence of Accelerometer Placement and/or Heart Rate on Energy Expenditure Prediction during Uphill Exercise. *J Mot Behav*. 2018; **50**(2): 127-33.

53. Trapnell, C, Roberts, A, Goff, L*, et al.* Differential gene and transcript expression analysis of RNA-seq experiments with TopHat and Cufflinks. *Nat Protoc*. 2012; **7**(3): 562-78.

**
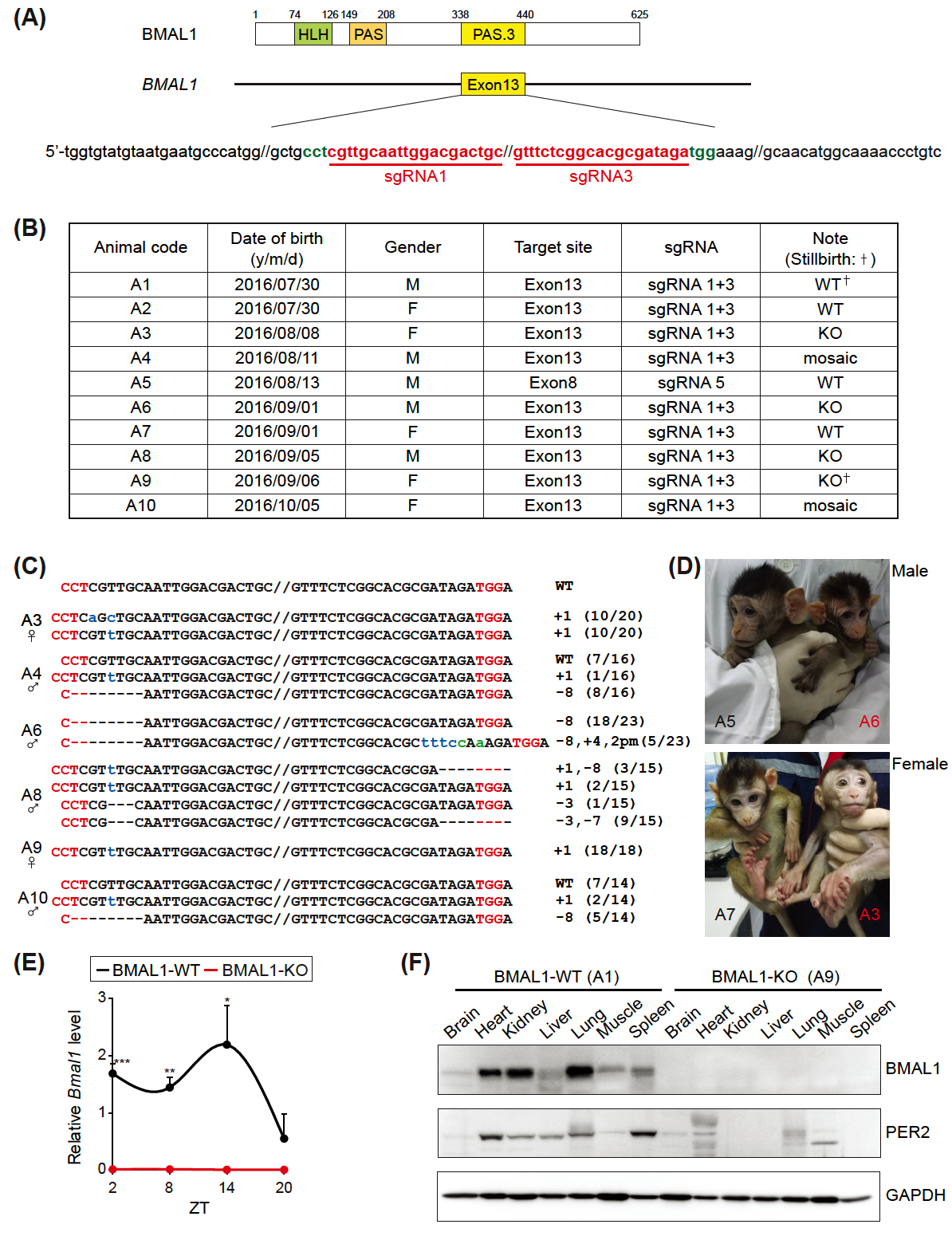
**

**Figure 1.** Generation of BMAL1 knockout cynomolgus monkey. (A) Designated PAS.3 domain and the respective sequences in *BMAL1* *exon*13 that were targeted in cynomolgus monkey. Red, sgRNA1 and 3 sequences. (B) Summary of all *BMAL1*-edited monkeys in this study. (C) Sequence alterations in the five CRISPR/Cas9-edited mutant monkeys, examined via PCR amplification of *BMAL1* *exon*13, followed by sequence analysis. (D) Images of male A5 (BMAL1-WT), male A6 (BMAL-KO), female A7 (BMAL1-WT), and female A3 (BMAL1-KO) cynomolgus monkeys at 4-month of age. (E) Expression of wild-type *BMAL1* transcript over zeitgeber time (ZT) 2, 8, 14 and 20 for BMAL1-WT versus BMAL1-KO monkeys, shown by the average blood mRNA level. (F) Immunoblots of BMAL1 and PER2 levels in major tissues collected from stillbirths A1 and A9. GAPDH was used as the reference. ^🞱^*P* < 0.05, ^🞱🞱^*P* < 0.01, ^🞱🞱🞱^*P* < 0.001; Student’s *t*-test.

~~
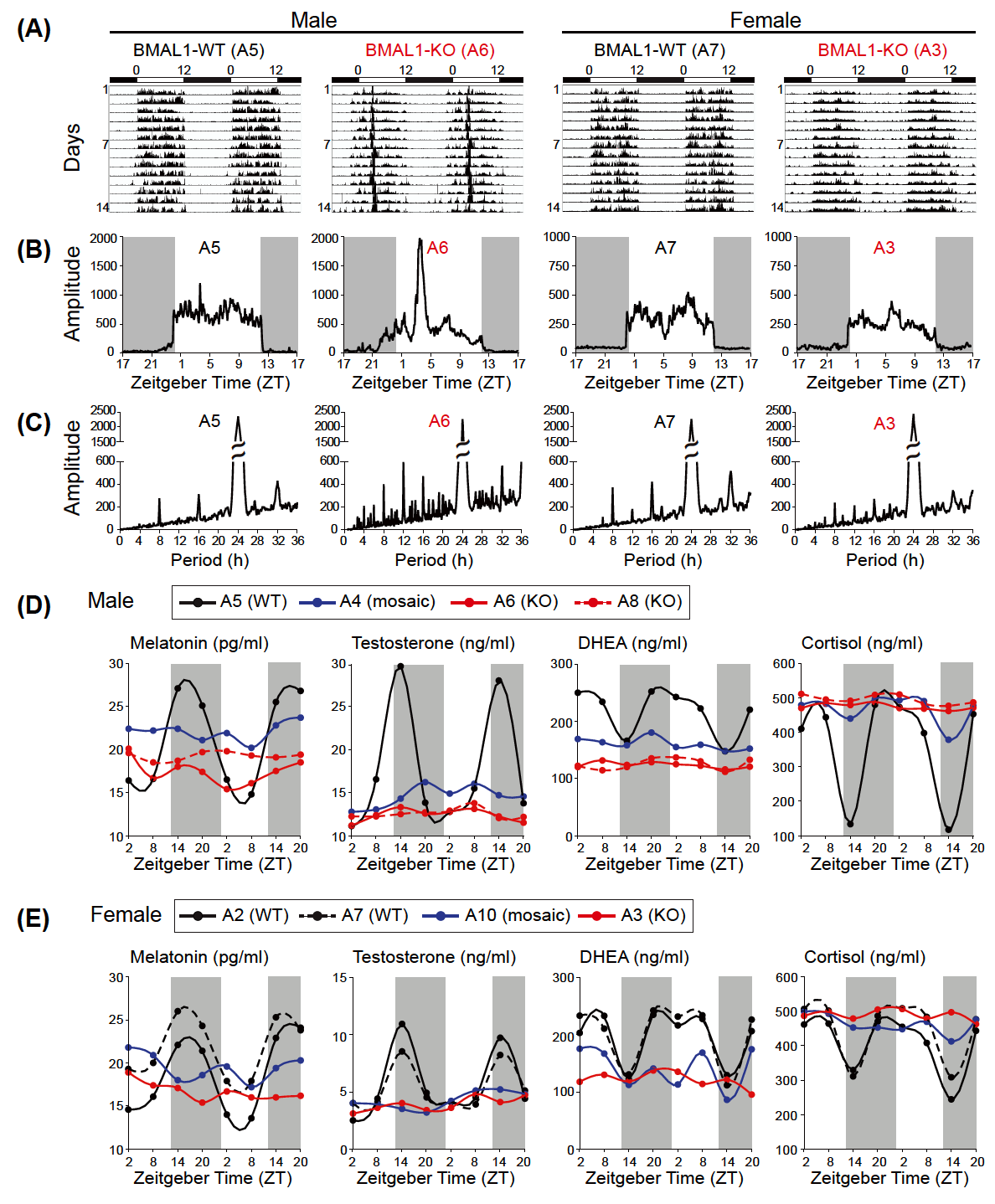
~~

**Figure 2.** Dysregulated circadian locomotor activities and hormonal levels in BMAL1-deficient monkeys. (A) Continuous 14-day locomotor activity recorded in BMAL1-WT and BMAL1-KO monkeys. Example actograms of A5, A6, A7 and A3 were shown under 12-hour light / 12-hour dark (L/D) conditions. (B)(C) Activity amplitude and chi-squared periodogram of the 14-day actograms. (D) Levels of melatonin, testosterone, DHEA and cortisol were assayed for monkey plasma samples obtained from temporal collections at 6-hour intervals over 48 hours. Results shown were from the male cohort. (E) Female hormonal assays in the same manner as in (D).

**
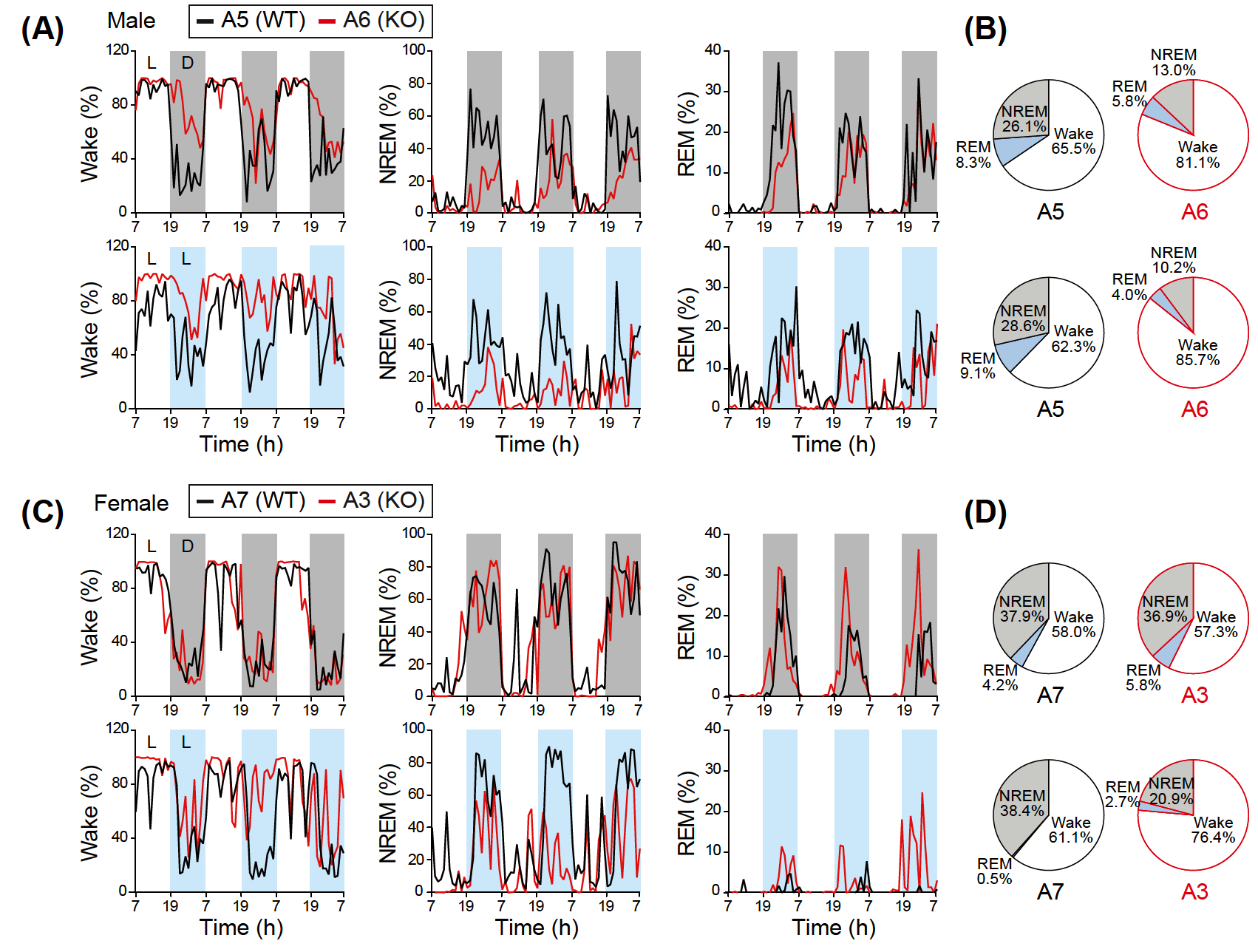
**

**Figure 3.** Altered sleep states and diurnal EEG oscillations in BMAL1-KO monkeys. (A) Telemetric EEG recoding in male BMAL1-WT (A5) and BMAL1-KO (A6) monkeys under 3-day L/D cycles (upper panels), or 3-day L/L cycles (lower panels). Stage scores for the proportions of wake, NREM, and REM states were shown. (B) Pie charts presented the averaged proportions of the three stages over the 3-day recording period in male monkeys. (C) Telemetric EEG recoding in female BMAL1-WT (A7) and BMAL1-KO (A3) monkeys under 3-day L/D cycles (upper panels), and 3-day L/L cycles (lower panels). (D) Pie charts were presented as in (B) for female monkeys.

**
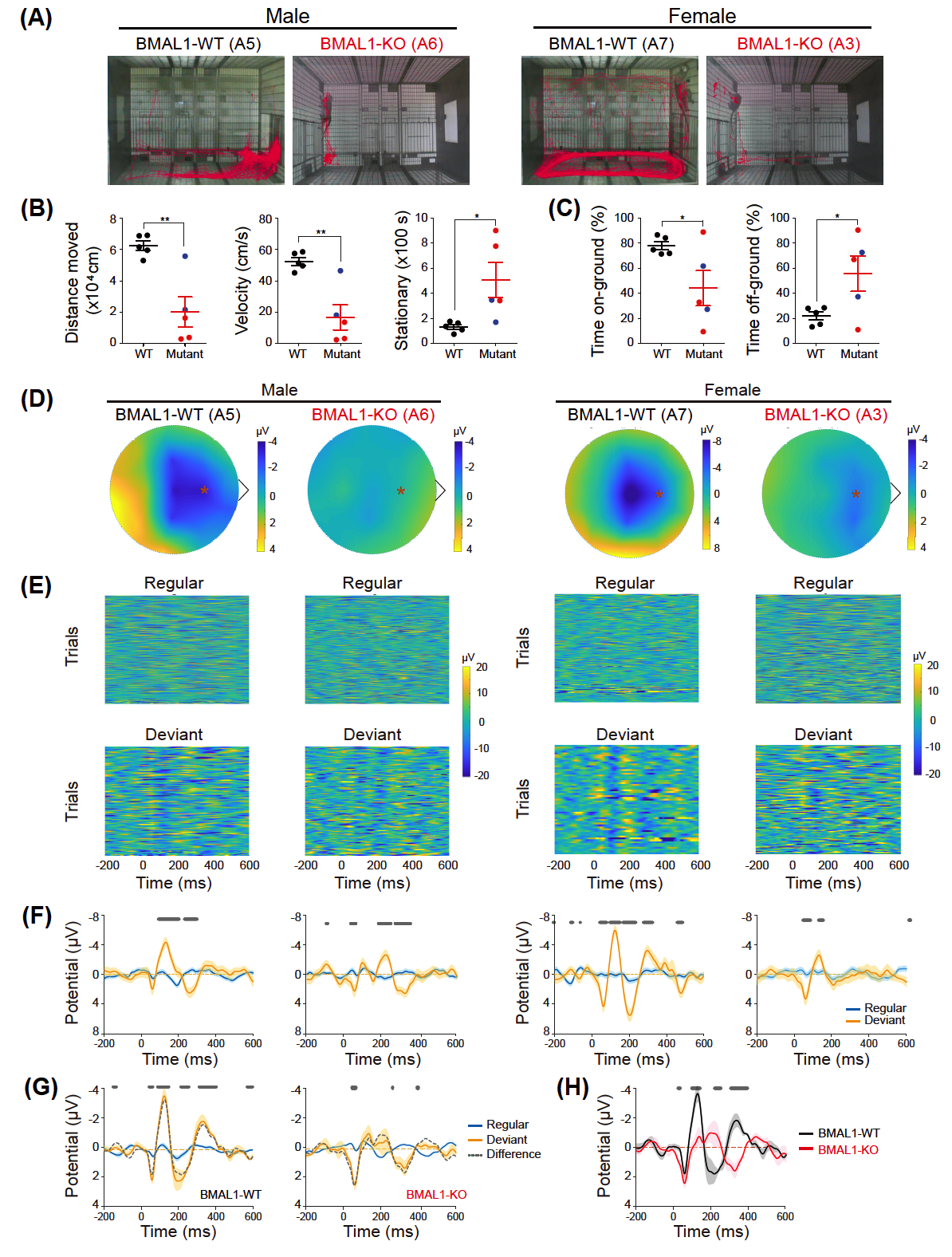
Figure 4.** Depression-related behavior and sensory processing impairment in BMAL1-KO monkeys. (A) Activity tracks (marked red) obtained by 20-minute videotape recordings for all BMAL1-edited and wild-type monkeys. (B) Summary of locomotor activities, including total distance, moving velocity, stationary time, and (C) time spent on-ground versus time spent off-ground were analyzed. In black, blue and red indicated BMAL1-WT, BMAL1 mosaic, BMAL1-KO monkeys, respectively. Data also included two wild-type age-matched male monkeys, in addition to the 8 monkeys listed in Fig. 1B. ^🞱^*P* < 0.05, ^🞱🞱^*P* < 0.01; Student’s *t*-test. (D) Topographic voltage maps of BMAL1 WT and KO monkeys. Asterisk indicates Fz electrode position. (E) Heat maps of voltage amplitudes induced by regular or deviant sound trials. ERP at Fz electrode were analyzed. (F) Average ERP summarized from (E). (G) MMN of BMAL1 WT and KO monkeys, as indicated by the differences between regular and deviant in dashed lines. (H) MMN of BMAL1-WT versus BMAL-KO monkeys summarized from (G). Significant differences were indicated with star labels on top (*P*<0.05, Student’s *t*-test), and SEMs were shown with shadowed areas.

**
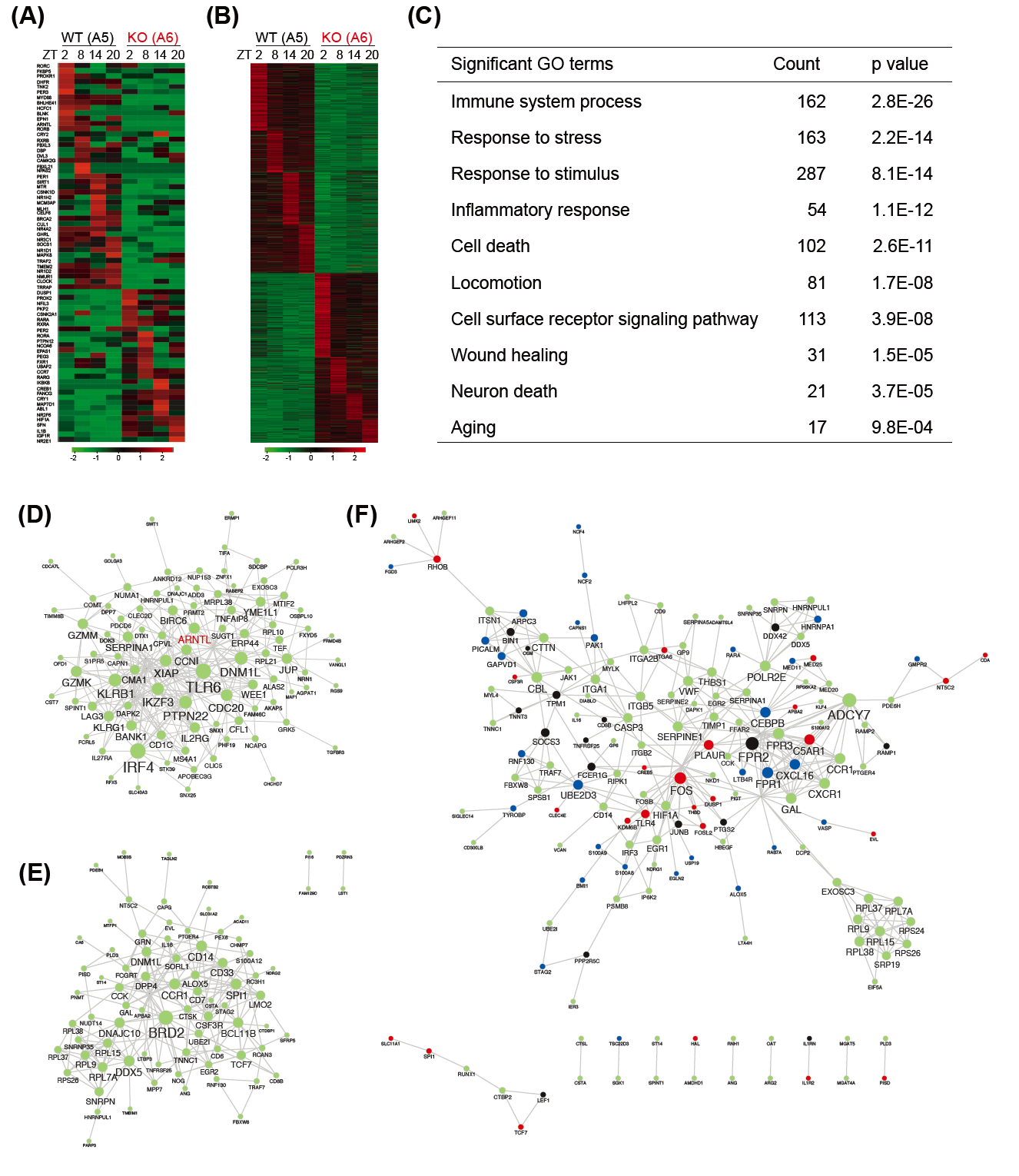
**

**Figure 5.** Blood transcriptome analysis of BMAL1-KO monkeys. (A) Representative examples of the expression levels of circadian controlled genes over time points ZT2, ZT8, ZT14 and ZT20. Results from male BMAL1-WT (A5) and BMAL1-KO (A6) monkeys were shown. (B) Heat maps of genes that were up- and down-regulated in their expression over time points ZT2, ZT8, ZT14 and ZT20, for monkeys A5 and A6. Results of greater than 2-fold changes were shown. (C) Gene Ontology (GO) terms for up-regulated genes shown in the transcriptome analysis in (B). (D)(E) STRING assembly for functional network in A5 (D, upper panel) and A6 (E, lower panel) at time point ZT2. The result showed that the network assembly became distinctly different between wild-type and BMAL1-KO monkeys (BMAL1 is labeled as “ARNTL” in the network). (F) Over-representing STRING network assembly of up-regulated targets in BMAL1-KO A6. Targets related to sleep deprivation (GSE39445) and major depressive disorder (GSE76826) are shown in black and blue, respectively, and those related to both were shown in red.
